# Altered TFEB subcellular localization in nigral neurons of subjects with incidental, sporadic and *GBA*-related Lewy body diseases

**DOI:** 10.1007/s00401-024-02707-z

**Published:** 2024-04-06

**Authors:** Tim E. Moors, Martino L. Morella, Cesc Bertran-Cobo, Hanneke Geut, Vinod Udayar, Evelien Timmermans-Huisman, Angela M. T. Ingrassia, John J. P. Brevé, John G. J. M. Bol, Vincenzo Bonifati, Ravi Jagasia, Wilma D. J. van de Berg

**Affiliations:** 1 Section Clinical Neuroanatomy and Biobanking, Department of Anatomy and Neurosciences, Amsterdam UMC, Vrije University, Amsterdam, The Netherlands; 2 Amsterdam Neuroscience, Program Neurodegeneration, Amsterdam, The Netherlands; 3 Roche Pharma Research and Early Development; Neuroscience and Rare Diseases Discovery and Translational Area, Roche Innovation Center, Basel, Switzerland; 4 Erasmus MC, Department of Clinical Genetics, University Medical Center Rotterdam, Rotterdam, The Netherlands

**Keywords:** Parkinsons disease, Alpha-synuclein, Lewy body diseases, TFEB, GBA, Lysosome

## Abstract

Transcription factor EB (TFEB) is a master regulator of genes involved in the maintenance of autophagic and lysosomal homeostasis, processes which have been implicated in the pathogenesis of *GBA*-related and sporadic Parkinson’s disease (PD), and dementia with Lewy bodies (DLB). TFEB activation results in its translocation from the cytosol to the nucleus. Here, we investigated TFEB subcellular localization and its relation to intracellular alpha-synuclein (aSyn) accumulation in *post-mortem* human brain of individuals with either incidental Lewy body disease (iLBD), *GBA*-related PD/DLB (*GBA*-PD/DLB) or sporadic PD/DLB (sPD/DLB), compared to control subjects. We analyzed nigral dopaminergic neurons using high-resolution confocal and stimulated emission depletion (STED) microscopy and semi-quantitatively scored the TFEB subcellular localization patterns. We observed reduced nuclear TFEB immunoreactivity in PD/DLB patients compared to controls, both in sporadic and *GBA*-related cases, as well as in iLBD cases. Nuclear depletion of TFEB was more pronounced in neurons with Ser129-phosphorylated (pSer129) aSyn accumulation in all groups. Importantly, we observed previously-unidentified TFEB-immunopositive perinuclear clusters in human dopaminergic neurons, which localized at the Golgi apparatus. These TFEB clusters were more frequently observed and more severe in iLBD, sPD/DLB and *GBA*-PD/DLB compared to controls, particularly in pSer129 aSyn-positive neurons, but also in neurons lacking detectable aSyn accumulation. In aSyn-negative cells, cytoplasmic TFEB clusters were more frequently observed in *GBA*-PD/DLB and iLBD patients, and correlated with reduced *GBA* enzymatic activity as well as increased Braak LB stage. Altered TFEB distribution was accompanied by a reduction in overall mRNA expression levels of selected TFEB-regulated genes, indicating a possible early dysfunction of lysosomal regulation. Overall, we observed cytoplasmic TFEB retention and accumulation at the Golgi in cells without apparent pSer129 aSyn accumulation in iLBD and PD/DLB patients. This suggests potential TFEB impairment at the early stages of cellular disease and underscores TFEB as a promising therapeutic target for synucleinopathies.

**Supplementary Information:**

The online version contains supplementary material available at 10.1007/s00401-024-02707-z.

## Introduction

Parkinson’s disease (PD) and dementia with Lewy bodies (DLB) are neurodegenerative diseases pathologically defined by the presence of neuronal cytoplasmic and axonal inclusions, termed Lewy bodies (LBs) and Lewy neurites, in circumscribed regions of the brain [8]. LBs consist of a variety of membranous deposits and proteins but are mainly defined by the presence of accumulated and post-translationally modified alpha-synuclein (aSyn), most notably Ser129-phosphorylated (pSer129) aSyn [4, 38, 62, 67]. Under physiological conditions, aSyn turnover is regulated by intracellular degradation systems, such as the ubiquitin-proteasomal system and the autophagy-lysosomal pathway (ALP) [75, 78]. However, pathological forms of aSyn are not adequately degraded by these protein degradation mechanisms, leading to its accumulation in PD and DLB. Failure of the ALP has been observed in PD/DLB, a finding supported by genome-wide association studies (GWAS) and linkage studies which have identified many genetic risk factors among ALP-related genes [20, 36, 54].

The most common genetic risk factors associated with PD and DLB are heterozygous mutations in the *GBA* gene. *GBA* encodes for the enzyme β-glucocerebrosidase (GCase), a lysosomal enzyme that catalyzes the hydrolysis of the sphingolipid glucosylceramide into glucose and ceramide [41, 57, 64]. Mutations in *GBA* can lead to reduced enzymatic activity causing the accumulation of its substrates. The exact mechanism linking *GBA* mutations, reduced GCase activity, or substrate accumulation to aSyn aggregation, as observed in PD, remains elusive. Pathological and experimental research has highlighted a complex, bidirectional relationship between GCase and aSyn, although the specifics of this interaction are not fully understood. In vitro evidence from nuclear magnetic resonance spectroscopy has identified a direct physical interaction between GCase and aSyn [80, 81]. Nonetheless, additional theories suggest their relationship might also be indirectly mediated through mechanisms such as increased GCase substrate levels [23, 63, 65, 70], decreased intracellular trafficking of GCase [34, 35, 65], and/or ALP dysfunction [7, 34, 35, 51]. Studies using induced pluripotent stem cell (iPSC)-derived neurons from patients with *GBA-*related PD showed widespread ALP impairment [6, 18, 58]. Overall, this research underscores the intricate interplay between GCase and aSyn in disease pathogenesis.

A key regulator of the ALP is transcription factor EB (TFEB). In homeostatic conditions TFEB is retained in the cytosol by phosphorylation, primarily by the inhibitory action of the mammalian target of rapamycin complex 1 (mTORC1) [31, 61]. The presence of stress stimuli—such as starvation [60], ER-stress [60] and oxidative stress [32]—result in mTORC1 inhibition and dephosphorylation of TFEB, inducing its translocation into the nucleus. Nuclear TFEB increases the expression of genes involved in the regulation of lysosomal, autophagic and retromer function, collectively called Coordinated Lysosomal Expression and Regulation (CLEAR) network, which include *GBA* and *TFEB* itself [14, 56, 61, 65, 82]. Results from a pioneering study by Decressac et al. implicated TFEB in synucleinopathies. Here, nuclear localization of TFEB was reduced in a rat model overexpressing aSyn and in postmortem midbrain brain tissue sections of PD donors [15]. Moreover, both overexpression and direct pharmacological activation of TFEB protect against accumulation of aSyn/tau and associated neurodegeneration in several murine and in vitro models [5, 16, 27, 66], suggesting TFEB could represent a potential therapeutic target for PD and other proteinopathies, such as DLB and Alzheimer’s disease [30, 33, 53, 66].

In this study, we investigated the relevance of TFEB subcellular distribution and its relation to the presence of aSyn cytopathology in early (iLBD) and late stages PD/DLB, both sporadic (sPD/DLB) and *GBA*-related (*GBA*-PD/DLB). To this end, we explored the subcellular localization of TFEB in dopaminergic neurons in the substantia nigra pars compacta (SNpc) of *post-mortem* human brains using high-resolution confocal and stimulated emission depletion microscopy (STED) microscopy, accounting for the presence or absence of intracellular pSer129 aSyn deposition. We included *post-mortem* midbrain tissue of iLBD patients (*N* = 3), PD/DLB patients with *GBA* variants (*N* = 10), sporadic PD/DLB patents (*N* = 9) and control subjects (*N* = 7). iLBD cases were included as early stages of Lewy body disease [19].

Our findings showed reduced nuclear localization of TFEB in sPD/DLB in SNpc dopaminergic neurons, supporting previous findings [15], particularly in neurons with pSer129 aSyn cytopathology. Additionally, we observed an unanticipated clustering of TFEB, which localized at the Golgi apparatus. The clustering was more frequent in the sPD/DLB group compared to controls and most severe in patients carrying *GBA* risk variants. TFEB clustering was also increased in iLBD cases compared to controls. Remarkably, the increase in TFEB clusters was observed both in cells without pSer129 aSyn cytopathology and—more prominently—in neurons with intracellular pSer129 aSyn. The TFEB immunopositive clusters did not colocalize with intracellular aSyn aggregates. Semi-quantitative measurement of the TFEB clusters in aSyn-negative cells was associated with reduced total GCase enzymatic activity and with increased Braak LB stage. Our results support a role of aberrant cytoplasmic TFEB localization in the early stages of cellular disease pathogenesis in sporadic and *GBA*-PD/DLB, and suggest its involvement in the early stages of aSyn accumulation.

## Materials and methods

### Post-mortem human midbrain tissue

*Post-mortem* human brain tissue was obtained from neuropathologically verified donors with iLBD (3 individuals), clinically diagnosed and neuropathologically confirmed sporadic PD or DLB without *GBA* variants (9 individuals), PD or DLB patients with *GBA* risk variants PD (9 individuals) and 15 age-matched control subjects without *GBA* risk variants. These patients represent a subset of a cohort for which the genotyping and various biochemical measurements were previously published [23, 39]. The demographics and group characteristics of the selected patients are presented in Table 1 and Suppl. Table 1 (online resource). The PD/DLB, *GBA*-PD/DLB and Control groups did not differ significantly in age at death. The PD/DLB and *GBA*-PD/DLB group did not differ significantly in age at disease onset and disease duration. *GBA* severe variants were identified according to their association with Gaucher disease (GD) type II or III, corresponding to Parlar et al*.* using the *GBA*-PD browser [45, 46], when available (Suppl. Tab. 1, online resource). One patient with multiple *GBA* variants (p.Asp140His, p.Glu326Lys, and p.Thr369Met) was classified as severe.

**Table 1:** Group characteristics of controls, iLBD, sPD/DLB and *GBA*-PD/DLB patients in the present study

	Controls (*N* = 7)	iLBD (*N* = 3)	sPD/DLB (*N* = 9)	*GBA*-PD/DLB (*N* = 9)
Age of death (years; mean ± SD)	77 ± 5	92 ± 6	77 ± 4	77 ± 6
Sex (M/F)	1/6	1/2	7/2	5/4
Postmortem delay (minutes; mean ± SD)	342 ± 96	336 ± 84	330 ± 60	330 ± 90
Braak Lewy body stage	0–1 (6/1)	3 (3)	4–6 (2/3/4)	4–6 (2/2/5)
Braak stage for neurofibrillary tangles	1–2 (2/5)	2–3 (2/1)	0–2 (3/4/2)	1–3 (8/0/1)
CERAD amyloid plaque score	O-B (1/4/2)	O-A (2/1)	O-B (4/2/3)	O-C (4/2/2/1)
Thal phase	0–2 (1/2/3)	0–1 (2/1)	0–3 (4/0/1/2)	0–4 (4/1/2/1/1)
Age of onset (years; mean ± SD)	–	–	64.9 ± 7.4	64.8 ± 9.1
Disease duration (years; mean ± SD)	–	–	11.7 ± 6.4	11.8 ± 6.9
Total GCase enzymatic activity in SN (pmol/min/mg; mean) [39]	1032 ± 142	–	828 ± 116	553 ± 209

Following all ethical and legal guidelines, informed consent for brain autopsy, the use of brain tissue, and clinical information for scientific research was given by either the donors or their family. Brains were dissected in compliance with standard operating protocols of the Netherlands Brain Bank and Brain Net Europe, and neuropathology was assessed by an experienced neuropathologist, according to the guidelines of Brain Net Europe [2, 3]. All donors were selected based on limited concomitant AD pathology (Braak neurofibrillary tangle stage ≤ 3 and CERAD ≤ B) and without micro-infarcts. For the groups of patients with iLBD, we selected cases with Braak LB stages 2 or 3, while the PD/DLB diagnostic group was selected with a Braak LB stage ≥ 4 [8]. Additionally, control subjects where selected based on the absence of significant aSyn pathology (Braak LB stage ≤ 1), no record of neurological disorders and lack of any *GBA* risk variant. Postmortem delay (PMD) for all donors was less than 10 h. Formalin-fixed paraffin-embedded (FFPE) tissue blocks of the midbrain containing the SN from all included donors were cut into 10 μm thick sections, which were used for immunohistochemistry (IHC) and multiple labelling experiments. Frozen SN and medial frontal gyrus (MFG) tissue was used for the messenger RNA (mRNA) quantification and GCase enzymatic activity quantification. For this, the tissue was pulverized frozen in pre-cooled stainless-steel grinding jars using a cryogenic grinder for 2 min or until full pulverization (30 Hz, Mixer Mill MM400, Retsch, Haan, Germany).

### Immunohistochemical staining procedure

Double and triple labeling experiments were performed on 10 μm thick FFPE sections. After deparaffinization, antigen retrieval (AR) was carried out using 10 mM citrate buffer (pH 6) in a steamer at 96 °C for 30 min. For certain antibodies, this was followed by a 10 min incubation in 100% formic acid. The AR used for each antibody is reported in Suppl. Tab. 2 (online resource). In double labelling protocols, sections were subsequently incubated in a blocking buffer (BB) containing 2% normal donkey serum (# 017–000-121, Jackson Immunoresearch) and 0.1% Triton-X in TBS (pH 7.6) for one hour, after which primary antibodies were incubated overnight at 4 °C. Afterwards, the sections were washed and incubated in appropriate secondary antibodies diluted in BB for 2 h at room temperature. In case of multiple labelling experiments, after incubation with secondary antibodies sections were blocked for 1 h in 5% normal rabbit serum (# 011–000-120, Jackson Immunoresearch) and 5% normal mouse serum (# 015–000-120, Jackson Immunoresearch) in TBS, and incubated for 2 h at room temperature in BB containing directly labelled antibodies [38]. Direct labelling of antibodies was performed using a Zenon™ Alexa Fluor 594 Rabbit IgG Labelling Kit (# Z25307, Thermo Fisher Scientific). DAPI was added to the BB at a concentration 1 µg/ml in the last incubation step. Sections were mounted in Mowiol mounting solution using glass cover slips (Art. No.: 630–2746; Glaswarenfabrik Karl Hecht, Sondheim, Germany). Negative control stainings, lacking primary antibodies, were performed to control for background/autofluorescence levels and nonspecific staining (Suppl. Figure 1, online resource). Single labellings for each antibody included in multiple-labelling experiments were carefully examined to control whether immunoreactivity patterns were caused by possible cross-reactivity between antibodies. The specificity of the TFEB antibodies used in this study was confirmed by Western Blot (WB) (Suppl. Figure 2b, online resource) [66, 71]. The antibodies utilized for the experiments of this study are summarized in Suppl. Table 2 (online resource).

### Confocal and stimulated emission depletion microscopy

Confocal laser scanning microscopy (CSLM) and stimulated emission depletion (STED) microscopy were performed using a Leica TCS SP8 STED 3X microscope (Leica Microsystems). All images were acquired using a HC PL APO CS2 100 × 1.4 NA oil objective lens, with the resolution set to a pixel size of 20 nm × 20 nm. Gated hybrid detectors were used in counting mode. Sections were sequentially scanned for each fluorophore, by irradiation with a pulsed white light laser at different wavelengths. A pulsed STED laser line at a wavelength of 775 nm was used to deplete the Alexa 594 fluorophore, while a continuous wave (CW) STED laser with wavelength of 592 nm was used to deplete the Alexa 488 fluorophore, respectively. After scanning, deconvolution was performed using CMLE (for confocal images) or GMLE (for STED images) algorithms in Huygens Professional software (Scientific Volume Imaging, Huygens, the Netherlands). All images were collected using the same settings, exposure and gamma during acquisition and adjusted for brightness/contrast in the same way using an ImageJ (National Institute of Health, USA) script prior to image analysis.

### Single-cell scanning

Images of neuromelanin-containing cells in the SNpc were collected using a 100 × 1.4 NA oil objective lens. The neuromelanin-containing portion of the cytoplasm was defined for each neuron using brightfield scans. In addition, selection of cells for scanning was based on the visibility of a nucleus and a defined cytoplasmic portion not filled with neuromelanin granules in the same z-plane. The presence of somatic pSer129 aSyn immunoreactivity was used to distinguish between cells with and without cytopathology. TFEB reactivity patterns were not used as a criterion for selection. For each cell, a z-stack of 1.05 µm was scanned based on the z-position of the nucleus (and when possible, nucleolus) using a z-step size of 0.15 µm. A total of 441 neuromelanin-containing dopaminergic cells was scanned, including 100 neurons of age-matched control subjects, 105 of iLBD, 109 of sPD/DLB and 129 of *GBA*-PD/DLB patients (Suppl. Tab. 3, online resource). The number of imaged cells matching our defined inclusion criteria ranged from 7 to 39 per individual and was restricted by extensive loss of nigral neurons in PD/DLB patients. A distinction was made between neurons with and neurons without subcellular immunoreactivity for pSer129 aSyn. This was done according to the previously described detailed subcellular phenotypes of pSer129 aSyn—including different cytoplasmic inclusions as well as cytoplasmic network. As these features were not observed in controls in a previous study by our group [38] or in the present study, they were therefore considered as highly specific for aSyn cytopathology.

### Semi-quantitative scoring and quantification of TFEB immunopatterns

For the semi-quantitative scoring of TFEB, raw confocal images were scanned using the same settings and processed in the same way in ImageJ. For each scanned cell, images from the brightfield, TFEB and DAPI channels were extracted and used for semi-quantitative analysis, without information about pSer129 aSyn. Subsequently, all images were blinded for diagnosis by randomized coding to ensure objectivity and nuclear TFEB reactivity was semi-quantitatively assessed by two independent raters. Based on pilot measurements in a subset of cells, the following scores were defined: 1—a pronounced lower density of punctate TFEB in the nucleus versus cytoplasm; 2—comparable densities of punctate TFEB in the nucleus versus cytoplasm; 3—pronounced nucleolar labeling. The inter-rater reliability of the nuclear TFEB scoring, estimated by the Cronbach’s alpha coefficient, was 0.88. An unexpected feature of TFEB immunoreactivity patterns was the presence of cytoplasmic clusters, seemingly independent of cytoplasmic TFEB punctae (discussed in results section). A TFEB cluster was defined as the presence of more than 0.7 µm of TFEB-positive structure in any direction in the raw CLSM images. This feature was semi-quantitatively scored in the same images by two independent raters as follows: 0—no clusters, 1—low (1–2- clusters), 2—intermediate (< 10 clusters), 3—severe (> 10 clusters). The inter-rater reliability of the TFEB cluster scoring, as estimated by the Cronbach’s alpha coefficient, was 0.91. Median cluster count per cell and median total cluster area per cell was quantified in all images with identical image processing and threshold settings using a custom-made ImageJ macro script. Each neuronal cell body was manually segmented in anonymized images based on brightfield and DAPI signal. A cut-off area of 0.5 µm^2^ was used to discriminate between TFEB cluster and punctae after particle recognition.

### RNA extraction and mRNA quantification

mRNA expression levels of selected CLEAR genes were analyzed using quantitative polymerase chain reaction (qPCR). For this purpose, total RNA was extracted from pulverized SN as well as MFG in a subset of cases, depending on tissue availability (see Suppl. Tab. 1, online resource). A Trizol/chloroform protocol for RNA extraction was used, as previously published [17]. To check for RNA quality, the purity of the extracted RNA was estimated by the absorbance ratio at 260/280 nm λ, using a Nanodrop spectrophotometer (Thermo Fischer Inc., Waltham, MA, USA). RNA integrity was calculated based on the 28S to 18S rRNA ratio using the RNA 6000 Nano Kit and the Agilent 2100 Bioanalyzer (Agilent Technologies Inc., Santa Clara, CA, USA) and was expressed as RNA integrity number (RIN). Only samples with RIN values ≥ 5.0 and PMD < 10 h were included in the analysis. SN samples had RIN values between 5.0 and 7.9 (mean = 6.0, variance = 0.48) and MFG samples between 5.6 and 8.0 (mean = 6.6, variance = 0.46). From the extracted total RNA samples, cDNA was synthetized using the High-Capacity cDNA Reverse Transcription Kit (Applied Biosystems, cat. n. 4,368,814, Thermo Fischer Inc., Waltham, MA, USA). Negative controls lacking the RT enzyme were included in the procedure to control for genomic DNA contamination during qPCR.

Intron-spanning primers were designed for the selected genes encoding for galactosylceramidase (*GALC*), hexosaminidase subunit alpha (*HEXA*), ß-glucocerebrosidase (*GBA*), microtubule associated protein 1 light chain 3 (*MAP1LC3A*), sequestosome 1 (*SQSTM1*), UDP-glucose ceramide glucosyltransferase (*UGCG*), VPS35 retromer complex component (*VPS35*) and for the reference genes pescadillo ribosomal biogenesis factor 1 (*PES1*), RNA polymerase II subunit A (*POLR2A*), ornithine decarboxylase antizyme 1 (*OAZ1*) and RNA polymerase II subunit F (POLR2F). Suitable TaqMan probes were selected from the Universal Probe Library (Roche Applied Science, Indianapolis, IN, USA). All primers used were purchased from Kaneka Eurogentec (Seraing, Belgium) and are listed in Suppl. Table 4 (online resource). Each cDNA sample was analyzed in triplicate (mean CV = 0.4%) and standard curves and efficiency values were calculated using the StepOne plus software (Thermo Fisher). The software geNorm (Genorm) was used to select the reference genes with the most stable expression among all cases. Results were quantified as relative expression normalized to the geometric mean [50, 74] of most suitable reference genes (PES1, POLR2A and POLR2F).

### GCase enzymatic activity quantification

The cases selected for this study are from a larger cohort whose bulk-tissue glucocerebrosidase activity was measured in [39] and the values relative to the subset of cases included in the present study were extracted. Briefly, pulverized MFG and SN tissue from a subset of cases were lysed in 50 mM sodium/phosphate, 150 mM NaCl buffer at pH 7.0, homogenized, sonicated and centrifuged as previously described [39]. Protein concentration was determined using a Bradford assay [9] and aliquots containing equal amounts of protein were incubated at 37 °C with 3 mM of the florigenic substrate 4‐methylumbelliferyl‐β‐D‐glucopyranoside (MUB-Glc) in equal amounts of McIlvaine reaction buffer (0.1 M citric acid, 0.2 M Na_2_HPO_4_), pH 4.5 with 0.2% taurodeoxycholate. After 60 min, the reaction was stopped with ice‐cold 0.2 M glycine‐NaOH buffer, pH 10.4 and the fluorescence of MUB released by enzymatic cleavage was measured with a FLUOstar Optima microplate reader (BMG Labtech, Ortenberg, Germany [λex/λem = 360 nm/450 nm]) [47]. Each sample was analyzed in triplicate alongside a negative control condition lacking the protein sample and an unbounded MUB standard curve. The enzymatic activity was calculated based on the MUB standard curve and expressed as amount of total protein necessary to hydrolyze 1 nmol of MUB-Glc in 1 min at 37 °C (pmol/min/mg). The enzymatic activities measured in the cases selected for this study showed a mean CV of 4.1% (variance = 3.3) and of 4.9% (variance = 4.3) for SN and MFG, respectively.

### Statistics and computing

To analyze the TFEB semi-quantitative scores, we applied Generalized Estimating Equations (GEE) models with an ordinal probit response for our statistical analysis to assure that our results could not be attributed to measurements in a single individual or to differences in the number of pathological cells per individual. For each test, Wald Chi-Square and B statistics (together with 95% confidence interval) are presented in the results section. For correlation analyses, either Pearson’s or Spearman’s tests were performed as required and as indicated in the results section. When comparing the normally distributed continuous data from the GCase enzymatic activity assay, one-way ANOVA with group as fixed factor and age as covariate was used to test for differences between the groups’ means, coupled with Games-Howell post-hoc test to identify specific differences between the groups. For the analysis of median TFEB cluster count and median total cluster area per cell, we used the Kruskal–Wallis test with Bonferroni-corrected pairwise comparisons. For the analysis of the mRNA quantification data, General Linear Models (GLM) were used with gene and disease group as fixed factors and age as covariate. All statistical analyses were performed using the software IBM SPSS statistics (version 26.0, IBM, Armonk, NY, USA). In all tests, the significance threshold was set a *p* ≤ 0.05. Graphs were generated using the software GraphPad Prism (version 8.2.1, GraphPad Software, San Diego, CA, USA). The graphical abstract (Fig. 7) was created using Biorender (BioRender.com, 2022).

## Results

### Description of TFEB immunoreactivity patterns in SNpc neurons

**Fig. 1:**
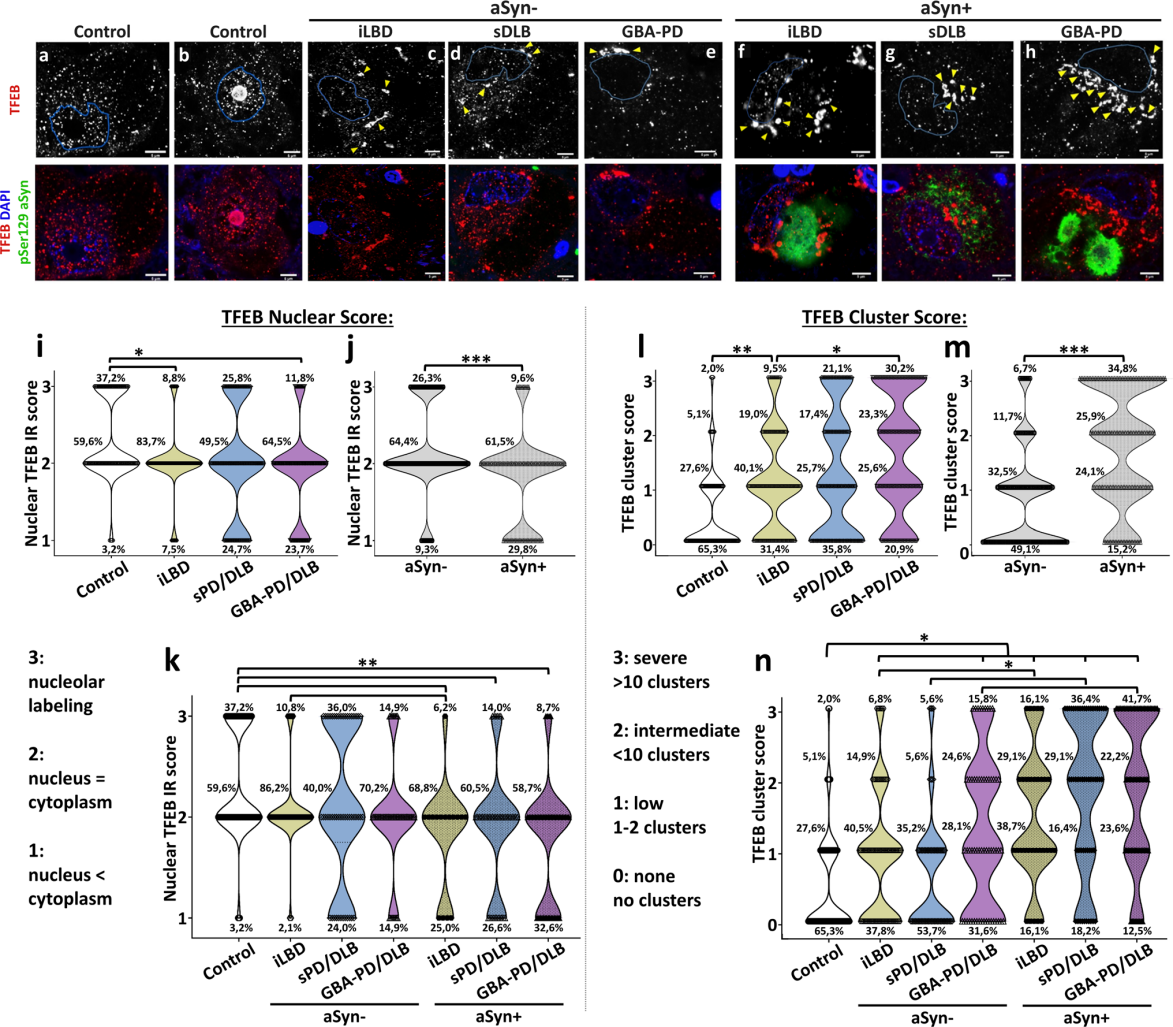
Reduced nuclear localization and increased perinuclear clustering of TFEB in iLBD, sPD/DLB and *GBA*-PD/DLB compared to controls in dopaminergic SN neurons with and without pSer129 aSyn cytopathology. **a**–**h** Representative raw confocal images of TFEB localization patterns in dopaminergic SNpc neurons in iLBD, PD/DLB patients and controls stained for TFEB (red) and pSer129 aSyn (green). **a**–**b** Neurons from control cases. **c**–**h** Neurons from diseased cases. **c**–**e** Neurons without pSer129 aSyn cytopathology; **f**–**h** Neurons with pSer129 aSyn cytopathology. Features immunoreactive for TFEB included nuclear and cytoplasmic punctae in different proportions, nucleolar labeling patterns (**b**), and perinuclear clusters (**c**–**h**). The outline of the DAPI signal is projected in blue. Yellow arrowheads indicate TFEB-immunopositive clusters. All scale bars represent 5 µm. **i**–**k** Nuclear TFEB is reduced in the disease groups compared to controls. **i** Frequency distributions of nuclear TFEB immunoreactivity (IR) semi-quantitative scores (as indicated). **j **Frequency distribution of nuclear TFEB IR scores in cells with and without subcellular pSer129 aSyn pathology across all diagnostic groups. **k** Frequency distributions of nuclear TFEB IR scores in cells with and without pSer129 aSyn pathology per group. Higher nuclear TFEB IR scores were more frequently found in controls compared to the iLBD, sPD/DLB and *GBA*-PD/DLB groups. **l**–**n **TFEB clusters are increased in the disease groups both in presence and absence of pSer129 aSyn cytopathology. **l** Frequency distributions for semi-quantitatively scored TFEB clusters (as indicated). **m **Frequency distribution of TFEB cluster scores in cells with and without subcellular pSer129 aSyn pathology across all diagnostic groups. **n** Frequency distributions of TFEB cluster scores in cells with and without pSer129 aSyn pathology per disease group. An increase in the TFEB cluster score was observed in iLBD, sPD/DLB and *GBA*-PD/DLB compared to controls, both in cells with and without pSer129 aSyn cytopathology. Within each disease group, TFEB clusters were observed more frequently in cells with pSer129 aSyn cytopathology compared to cells without pSer129 aSyn cytopathology. aSyn + : neurons with pSer129 aSyn cytopathology; aSyn-: neurons without pSer129 aSyn cytopathology. *p < 0.05; **p < 0.005; ***p < 0.001. TFEB antibody: Bethyl A303-673A

When analyzing TFEB immunostaining patterns using selected antibodies (Fig. 1a–h; Suppl. Figure 2–4, online resource) in the SNpc of iLBD, sporadic and *GBA*-related PD/DLB cases, we observed heterogeneous profiles and distribution patterns in neuromelanin containing dopaminergic neurons in all cases. Such heterogeneity could be observed among neurons of the same subject (Suppl. Figure 3, online resource).

#### -Nuclear and cytoplasmic TFEB punctae

Most prominently, immunopatterns were characterized by many TFEB-positive punctae, which were distributed throughout the cytoplasm as well as in the nucleus (Fig. 1a-h; Suppl. Figure 3, online resource). In a subset of these neurons, the nucleolar portion of the nuclear compartment—outlined based on DAPI signal—was strongly labeled by TFEB (Fig. 1b). Overall, the scanned neurons showed heterogeneity in the distribution of TFEB immunopositive punctae in the nucleus versus the cytoplasm ranging from neurons displaying high density of TFEB punctae in nucleus compared to cytoplasm (Fig. 1a,b), to neurons with very limited nuclear TFEB punctae (Fig. 1e,h).

#### -Somatic TFEB clusters

In addition to immunoreactive punctae (typically around 0.3 µm in the raw CSLM signal), a subset of neurons (particularly in the disease groups) displayed larger TFEB immunoreactive clusters in the perinuclear cytoplasm (Fig. 1c–h: yellow arrowheads; Suppl. Figure 3,4, online resource). These profiles appeared more frequently in neurons from diseased patients compared to controls and could be observed both in neurons with and without intracellular aSyn cytopathology. The frequency and number of the observed cytoplasmic clusters appeared variable among neurons and seemed to be increased in the presence of subcellular pSer129 aSyn immunoreactivity compared to neurons without apparent aSyn cytopathology (Fig. 1f–h). These clustered patterns were observed using different TFEB antibodies (Suppl. Figure 4, online resource).

To verify that the TFEB clusters observed were not the result of non-specific antibody binding in fixed tissue specimens and could be also observed in vitro, we differentiated human embryonic stem cells (hESC)-derived neurons from a healthy control case (wt) and an isogenically derived hESC line lacking *GBA* (*GBA* KO) to model for the reduced GCase activity observed in *GBA*-PD/PDD cases. After staining for TFEB and cytoskeletal protein MAP2, a CLSM analysis revealed the presence of a subset of neurons (both WT and *GBA*-KO) displaying perinuclear immunopositive TFEB clusters, similar to what observed in vivo (Suppl. Figure 2a-c, online resource). The frequency of cells with TFEB-positive clusters did not differ between the two lines. Interestingly, the analyzed neurons displaying TFEB clusters often displayed signs of cellular stress, such as altered cellular morphology, reduced nuclear shape and DNA condensation, suggesting this phenotype might be associated with cellular stress.

Subsequently, we aimed to determine if phosphorylated TFEB could be detected within the observed clusters, as phosphorylation is known to mediate TFEB's cytosolic retention by inhibiting its nuclear translocation [31, 61]. However, none of the tested antibodies (see Suppl. Tab. 2, online resource) revealed specific labeling of endogenous TFEB in postmortem tissue by IHC.

### Nuclear TFEB immunoreactivity is reduced in iLBD, sPD/DLB and GBA-PD/DLB

Nuclear TFEB immunoreactivity was semi-quantitatively scored in a blinded analysis to compare control cases and the different disease groups (see *Materials and methods*). The analysis showed a higher number of neurons with increased nuclear TFEB in controls compared to each of the diseased groups (*p* = 0.025; Wald Chi-Square: 4.995; *B* = 0.754; 95% confidence interval (CI) [− 1.415:0.093]). In particular, iLBD donors (*p* = 0.043, Wald Chi-Square: 4.093; *B* = − 0.665; 95% CI [− 1.308: − 0.021]) and *GBA*-PD/DLB patients (*p* = 0.009; Wald Chi-Square: 6.750; *B* = − 0.927; 95% CI [− 1.627: − 0.228]) showed reduced nuclear TFEB immunoreactivity compared to controls, while the same effect was less pronounced in sPD/DLB cases with advanced Braak stages (*p* = 0.113; Wald Chi-Square: 2.513; *B* = − 0.669; 95%CI [− 1.496: 0.158]; Fig. 1i). These results are in support of previous indications that nuclear TFEB immunoreactivity is reduced in dopaminergic neurons in the SNpc of patients with PD compared to controls [15].

Interestingly, the analysis showed that nuclear localization of TFEB was reduced in neurons affected by pSer129 aSyn cytopathology compared to neurons without intracellular pSer129 aSyn immunoreactivity (Fig. 1j). When comparing neurons with and without pathology in the different diagnostic groups, we observed less nuclear immunostaining in cells with pSer129 aSyn pathology than in cells without (*p* = 0.0005; Wald Chi-Square: 11.959; *B* = − 0.720; 95% CI [− 1.128: − 0.312]). This effect was still significant when excluding controls from the analysis (*p* = 0.0013; Wald Chi-Square: 10.308; *B* = − 0.521; 95% CI [− 0.839: − 0.203]). While nuclear TFEB immunoreactivity in aSyn-positive neurons was reduced compared to controls in patients with either iLBD, sPD/DLB and *GBA*-PD/DLB (Fig. 1k), we observed a trend for lower nuclear immunoreactivity scores also in neurons without apparent aSyn pathology in the iLBD group (*p* = 0.104; Wald Chi-Square: 2.646; *B* = − 0.543; 95% CI [− 1.197: 0.111]) and in the *GBA*-PD/DLB group (*p* = 0.071; Wald Chi-Square: 3.253; *B* = − 0.705; 95% CI [− 1.470:0.061] compared to cells in control cases. This trend was not observed in sPD/DLB patients (*p* = 0.365; Wald Chi-Square: 0.821; *B* = − 0.465; 95% CI [− 1.472:0.541]).

### Cytoplasmic clustering of TFEB is increased in iLBD, sPD/DLB and GBA-PD/DLB

**Fig. 2:**
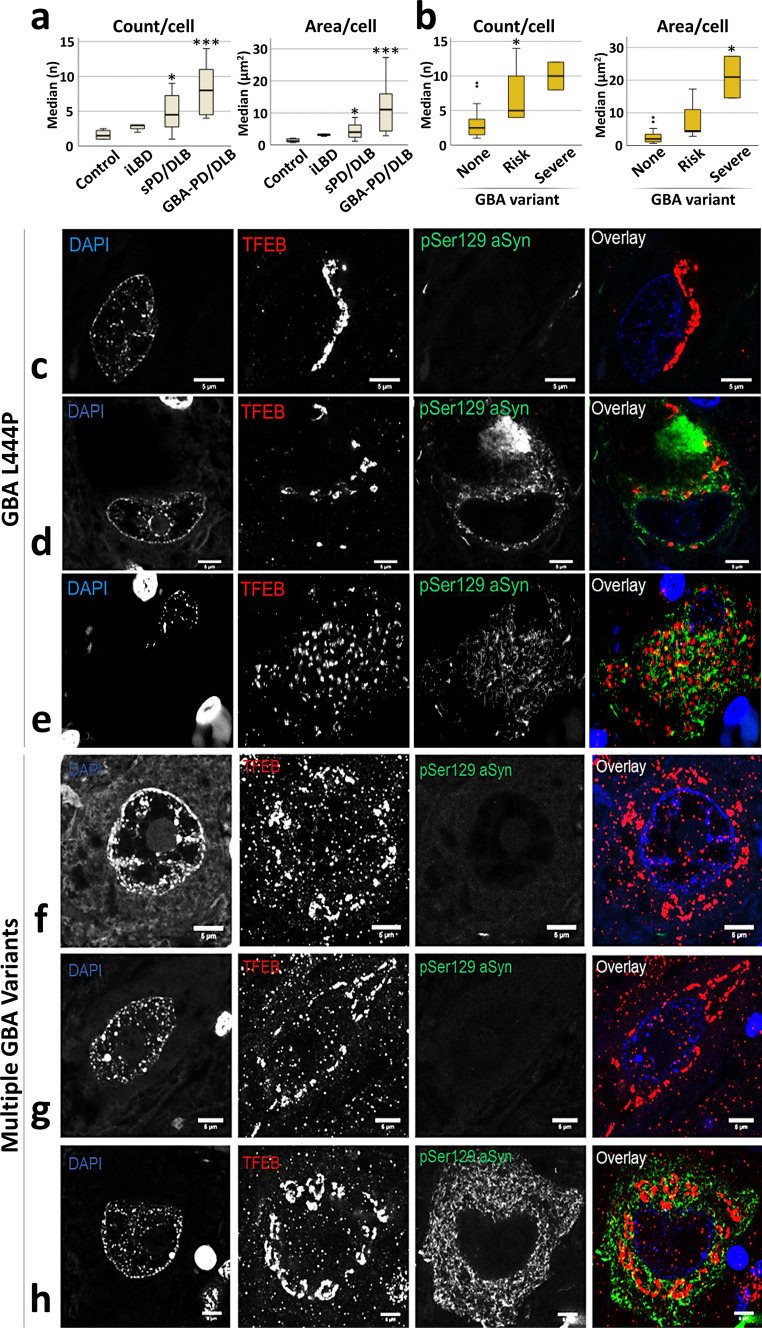
Representative TFEB immunoreactivity patterns in dopaminergic neurons in the SNpc of patients carrying severe *GBA* variants. **a**–**b** Quantification of median TFEB cluster count and total area per cell in cells with TFEB perinuclear clusters. **a** Comparison between study groups. **b** Comparison between *GBA* variants based on severity. **c**–**h** Representative confocal images of dopaminergic neurons of the SN stained for TFEB (red) and pSer129 aSyn (green) from donor ID26, carrying an L444P variant (**c**–**e**), and from donor ID24, which carries multiple *GBA* variants (**f**–**h**). TFEB immunoreactivity in neurons of PD patients with severe *GBA* variants was characterized by dense clusters in the cytosol, with limited immunopositive punctae in the cytoplasm and, particularly, in the nucleus. These patterns were not only observed in neurons with pSer129 aSyn pathology (**d**, **e**, **h**), but also in neurons without apparent aSyn pathology (**c, f, g**). All scale bars represent 5 µm. *p < 0.05; ***p < 0.005 vs Control/None. TFEB antibody: Bethyl A303-673A

We further conducted a semi-quantitative scoring of the TFEB clusters in the neuronal cytoplasm, which we defined as a ‘TFEB cluster score’. This analysis revealed pronounced differences between diagnostic groups, as the proportion of neurons displaying a higher TFEB cluster score was significantly increased in diseased patients compared to controls (*p* = 0.0002; Wald Chi-Square: 14.019; *B* = 1.034; 95% CI [0.493: 1.575]). This effect was significant in iLBD (*p* = 0.004; Wald Chi-Square: 8.218; *B* = 0.798; 95% CI [0.252: 1.343]), in sPD/DLB (*p* = 0.004; Wald Chi-Square: 8.227; *B* = 0.938; 95% CI [0.297: 1.580]) as well as in *GBA*-PD/DLB patients (*p* = 0.0003; Wald Chi-Square: 17.447; *B* = 1.336; 95% CI [0.709: 1.962]) compared to controls, with the *GBA*-PD/DLB group showing the strongest effect (Fig. 1l). Strikingly, the TFEB clusters were increased both in cells with and without aSyn cytopathology in patients versus controls (Figs. 1 and 2). In pSer129 aSyn-negative cells, the effect was especially pronounced in the *GBA*-PD/DLB group, reflected by an eightfold increase in the percentage of cells with severe TFEB clusters (score 3) compared to controls.

When comparing pSer129 aSyn-negative and pSer129 aSyn-positive cells, the TFEB cluster scores were particularly increased in neurons with pSer129 aSyn cytopathology (*p* = 2.38 E-12; Wald Chi-Square: 49.139; *B* = 1.092; 95% CI [0.787: 1.558], Fig. 1m) across all groups. The increase remained significant when excluding controls from the analysis (*p* = 8.77E-11; Wald Chi-Square: 42.078; *B* = 0.892; 95% CI [0.623: 1.162]).

However, our analysis showed an increase in the TFEB cluster scores also in cells without detectable pSer129 aSyn in the diseased groups compared to controls. Neurons negative for pSer129 aSyn showed significantly more TFEB clustering in iLBD (*p* = 0.016; Wald Chi-Square: 5.756; *B* = 0.649; 95% CI [0.119: 1.180]) and *GBA*-PD/DLB (*p* = 0.018; Wald Chi-Square: 5.572; *B* = 0.991; 95% CI [0.168: 1.814]) patients compared to controls (Fig. 1n), while a trend was observed in cells of sPD/DLB patients without cytoplasmic pSer129 aSyn immunoreactivity (*p* = 0.421; Wald Chi-Square: 0.647; *B* = 0.295; 95% CI [− 0.424: 1.013]).

### Cytoplasmic clustering of TFEB in patients with pathogenic GBA variants

Alterations in TFEB distribution were most pronounced in the group of *GBA*-PD/DLB patients compared to controls. To further explore the impact of *GBA* mutations on TFEB, we quantified the median TFEB cluster count and total TFEB cluster area per cell in cluster-positive cells (Fig. 2a, b). We identified an increased number of clusters and total cluster area in sPD/DLB and *GBA*-PD/DLB compared to controls (Fig. 2a). Notably, when looking at the presence and severity of *GBA* mutations, we observed an overall increase in cluster number and total area per cell in cases carrying *GBA* risk variants, which was further increased in cases carrying severe *GBA* variants compared to controls (Fig. 2b). To further investigate this observation, we selected two donors with severe *GBA* mutations to assess their TFEB immunoreactivity patterns in more detail. TFEB immunopatterns in the patients with severe pathogenic *GBA* variants (resulting in GCase deficiency) revealed extreme clustering in neuromelanin-containing dopaminergic neurons (Fig. 2c–h; Suppl. Video 1, online resource). The effect was observed both in cells with (Fig. 2d, e, h) and without pSer129 aSyn cytopathology (Fig. 2c, f, g). In these cells, TFEB immunoreactivity was concentrated in large cytosolic clusters showing limited additional immunopositive punctae in the cytoplasm and in the nucleus (Fig. 2c–h).

### TFEB clusters localize at the Golgi apparatus

**Fig. 3:**
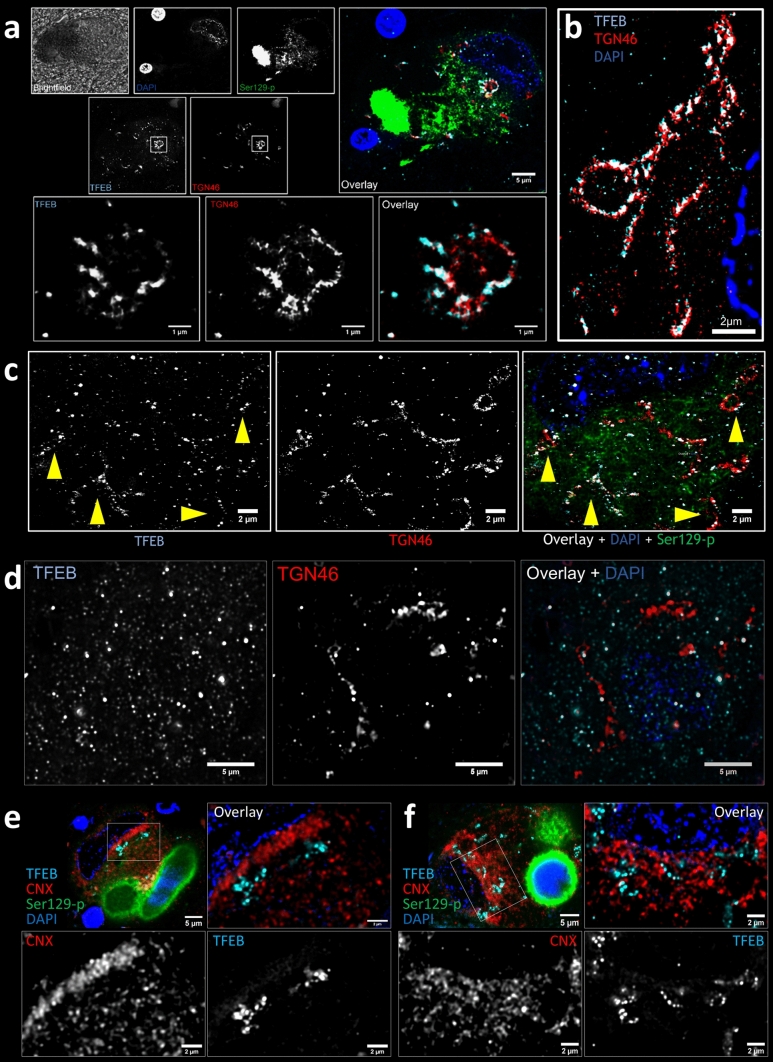
Cytoplasmic TFEB clusters localize at the Golgi in dopaminergic SNpc neurons. Deconvolved confocal (**a** upper panels, **d** and **e**–**f** upper-left panels) and STED (**a** lower panels, **b**, **c**,** e**–**f** zoom-ins) images. **a**–**d **Colocalization analysis between TFEB (cyan) and Golgi marker TGN46 (red). **a **Overview image (upper panels) and detailed magnification (lower panels) taken in sPD patient ID17 in a dopaminergic SNpc neuron with subcellular pSer129 aSyn pathology. **b **Image taken in a dopaminergic SNpc neuron with subcellular pSer129 aSyn pathology in *GBA*-PD patient ID24, demonstrating extensive perinuclear TFEB immunoreactivity localizing in the Golgi and displaying a detailed cisternal morphology. **c** Image taken in a DLB patient with a Glu326Lys variant (ID37) showing TFEB immunopositive punctae in the cytoplasm and partial colocalization to the Golgi (indicated by yellow arrowheads). **d **Image taken in a dopaminergic neuron of a control (ID9) without aSyn cytopathology, in which cytoplasmic TFEB immunopositive punctae show limited colocalization with TGN structures. **e**–**f** Colocalization analysis between TFEB (cyan) and ER marker Calnexin (CNX, red). Overview image (upper-left panel) and detailed magnification (other panels) taken in sPD patient ID26 (**e**) and ID39 (**f**) showing no colocalization between TFEB and TFEB clusters with the ER. Scale bars: **a** upper panels = 5 µm; lower panels = 1 µm; **b**–**c** 2 µm; **d** 5 µm; **e** upper-left panel = 5 µm, other panels = 2 µm. TFEB antibody: Bethyl A303-673A

TFEB immunopositive clusters were localized mainly at the perinuclear portion of the cytoplasm. We used STED microscopy to visualize the clusters in more detail and observed that the arrangement of TFEB within the clusters resembled a cisternal morphology. This observation led us to hypothesize that TFEB might cluster at the Golgi apparatus. This hypothesis was explored using a multiple labeling experiment with antibodies against pSer129 aSyn, TFEB, TGN46—a marker for the trans-Golgi network (TGN)—and DAPI (Fig. 3), combined with super-resolution STED microscopy. Our results demonstrated the localization of the TFEB clusters at the Golgi (Fig. 3; Suppl. Figure 1b, online resource). Where, in most neuromelanin-containing dopaminergic neurons, smaller TFEB clusters occupied a small part of the Golgi (Fig. 3a, c), in other cells extensive TFEB labelling was observed throughout the entire structure (Fig. 3b). In contrast, smaller TFEB punctae showed only limited colocalization with the Golgi, both in neurons with and without clusters (Fig. 3d). Colocalization of TFEB clusters at the Golgi was also confirmed using the cis-Golgi marker GOLGA2 (Suppl. Figure 5, online resource). No noteworthy colocalization was observed between the TFEB clusters and the endoplasmic reticulum (ER), investigated by staining for the ER marker Calnexin (Fig. 3e, f). Together, our STED observations suggest that TFEB accumulates at the Golgi under pathological conditions in sporadic and *GBA*-PD/DLB.

### TFEB clusters do not colocalize with aSyn

**Fig. 4:**
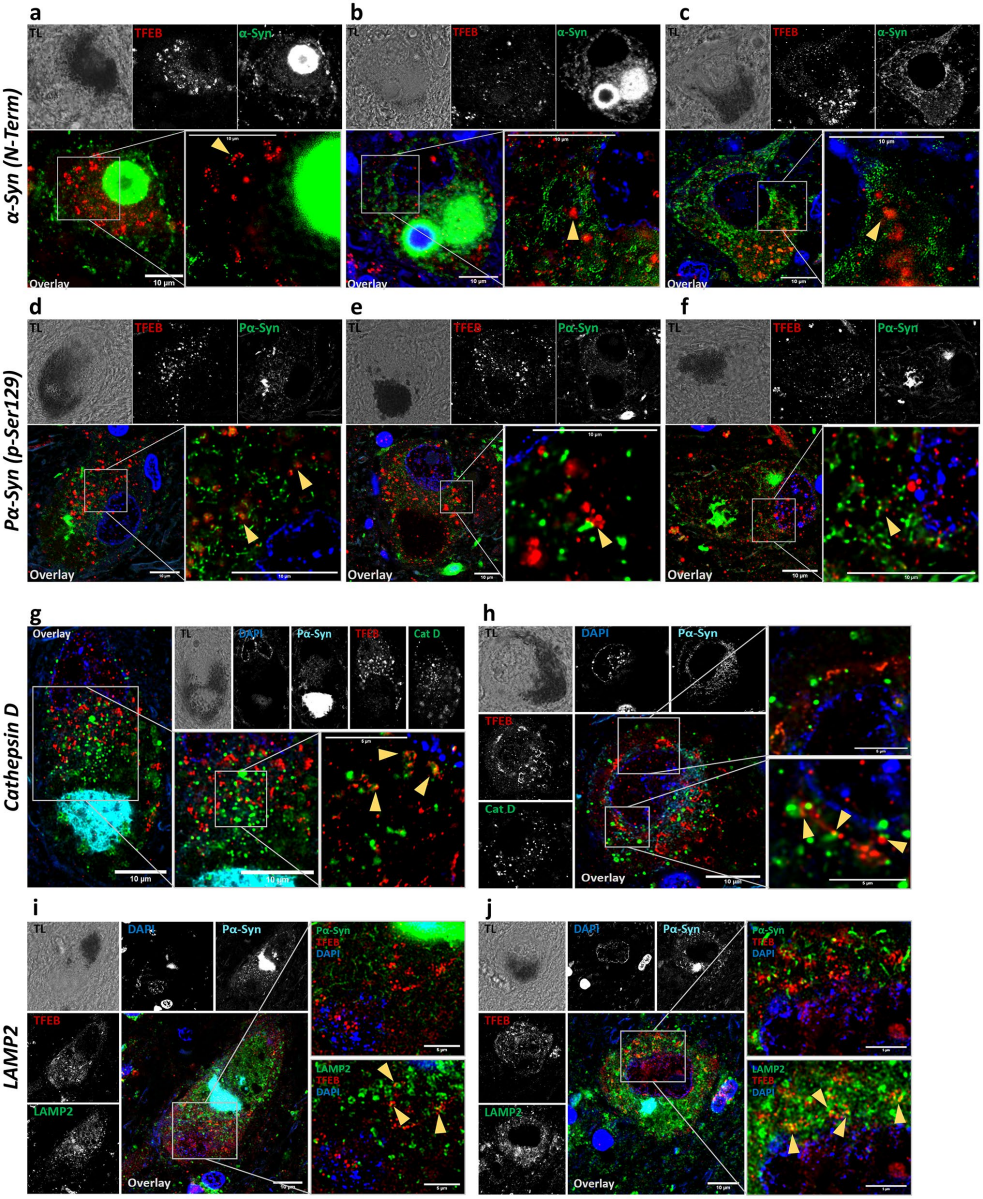
Cytoplasmic TFEB clusters partially colocalize with lysosomal markers, but not with aSyn. Representative transmitted light (TL, upper-left panels), deconvolved CLSM (central and right upper panels) and STED (lower-right panels) images of dopaminergic SNpc neuron from donor ID27 (sPD; **a**,** d**), ID37 (*GBA*-DLB; **b**,** e**) and ID16 (*GBA*-PD; **c**,** f**), ID43 (*GBA*-DLB; **g**), ID39 (sDLB; **h**), ID22 (sPD; **i**) and ID24 (*GBA*-PD; **j**). **a**–**f** Colocalization analysis of TFEB clusters with aSyn. No noteworthy colocalization was observed between TFEB clusters and aSyn, while limited colocalization was observed with TFEB puncta. **a**–**c** Staining for TFEB (red) and alpha-synuclein (N-19, N-Term, aa 1–60) (α-Syn, green) in neurons with intracellular LB (**a**), intracellular LB with amorphous aggregate (**b**) and diffuse intracellular staining (**c**) showing no colocalization with TFEB clusters. **d**–**f** Staining for TFEB (red) and Ser129-phosphorylated alpha-synuclein (PS129 α-Syn, green) in neurons with intracellular small amorphous aggregate (**d**, **f**) and intracellular diffuse staining (**e**) showing sporadic colocalization with TFEB clusters. **g**–**j** Colocalization analysis of TFEB clusters with lysosomal markers. **g**–**h** Staining for TFEB (red), Ser129-phosphorylated aSyn (Pα-Syn, cyan) and lysosomal protein Cathepsin D (Cat D, green) in neurons with intracellular amorphous aggregate (**g**) and diffuse intracellular staining (**h**). **i**–**j** Staining for TFEB (red), Ser129-phosphorylated aSyn (Pα-Syn, cyan/green as indicated in each panel) and lysosomal protein LAMP2 (LAMP2, green) in neurons with intracellular amorphous aggregate (**i**, **j**). The stainings showed colocalization points between TFEB and TFEB clusters with the lysosomal markers. Scale bars = 5/10 uM µm as indicated in each panel. TFEB antibody: Bethyl A303-673A

Altered subcellular TFEB localization was observed both within neurons with intracellular deposits of aSyn—being a LB, an amorphous aggregate or a diffuse intracellular staining—as well as within neurons without appreciable intracellular aSyn accumulation (Fig. 4a–f). Nonetheless, aSyn immunopositive neurons showed a higher number of clusters compared to aSyn-negative cells (Fig. 1). Interestingly, previous reports have described a potential protein–protein interaction between TFEB and aSyn [40, 43]. Thus, we investigated whether colocalization of TFEB and aSyn could be observed in SNpc neurons with intracellular aSyn deposition in sPD/DLB and *GBA*-PD/DLB cases. First, we stained for TFEB and for aSyn with an antibody directed towards the N-term portion of aSyn (aa 1–60, N-19), which is expected to recognize a substantial pool of non-truncated and truncated aSyn proteoforms (Fig. 4a–c) [73]. CLSM and STED microscopy showed no notable colocalization between TFEB clusters and aSyn (Fig. 4a–c). Analysis of TFEB and pS129 aSyn immunostaining revealed only sporadic colocalization points (Fig. 4d–f, arrowheads). Similar results were obtained with antibodies targeting different epitopes along the sequence of aSyn (Suppl. Figure 6, online resource). Interestingly, partial colocalization was observed between TFEB punctae and classical (ring-shaped) LBs (Suppl. Figure 6e, f, online resource), which was not observed for the larger TFEB clusters. Overall, these results demonstrate that TFEB clusters do not colocalize with pathological aSyn-positive morphologies, suggesting that TFEB clustering might not be primarily due to its interaction with aSyn aggregates.

### TFEB clusters show partial colocalization with lysosomal markers

The main regulation of TFEB happens at the lysosomal level, as functional TFEB transiently associates with the cytoplasmic side of the lysosomal membrane, where it undergoes phosphorylation. To investigate whether the TFEB observed in the perinuclear clusters retains the ability of localizing at the lysosome, we studied the colocalization of TFEB clusters with lysosomal markers by CLSM and STED microscopy (Fig. 4g–j). Staining for the lysosomal luminal protein Cathepsin D (CTSD) (Fig. 4g, h) and for the lysosomal membrane protein LAMP2 (Fig. 4i, j) showed the presence of colocalization points between TFEB clusters and lysosomes. The same was also observed for smaller TFEB punctae (Fig. 4g–j: arrowheads).

### Total GCase enzymatic activity and expression of selected CLEAR genes are reduced in sPD/DLB and GBA-PD/DLB compared to controls

**Fig. 5:**
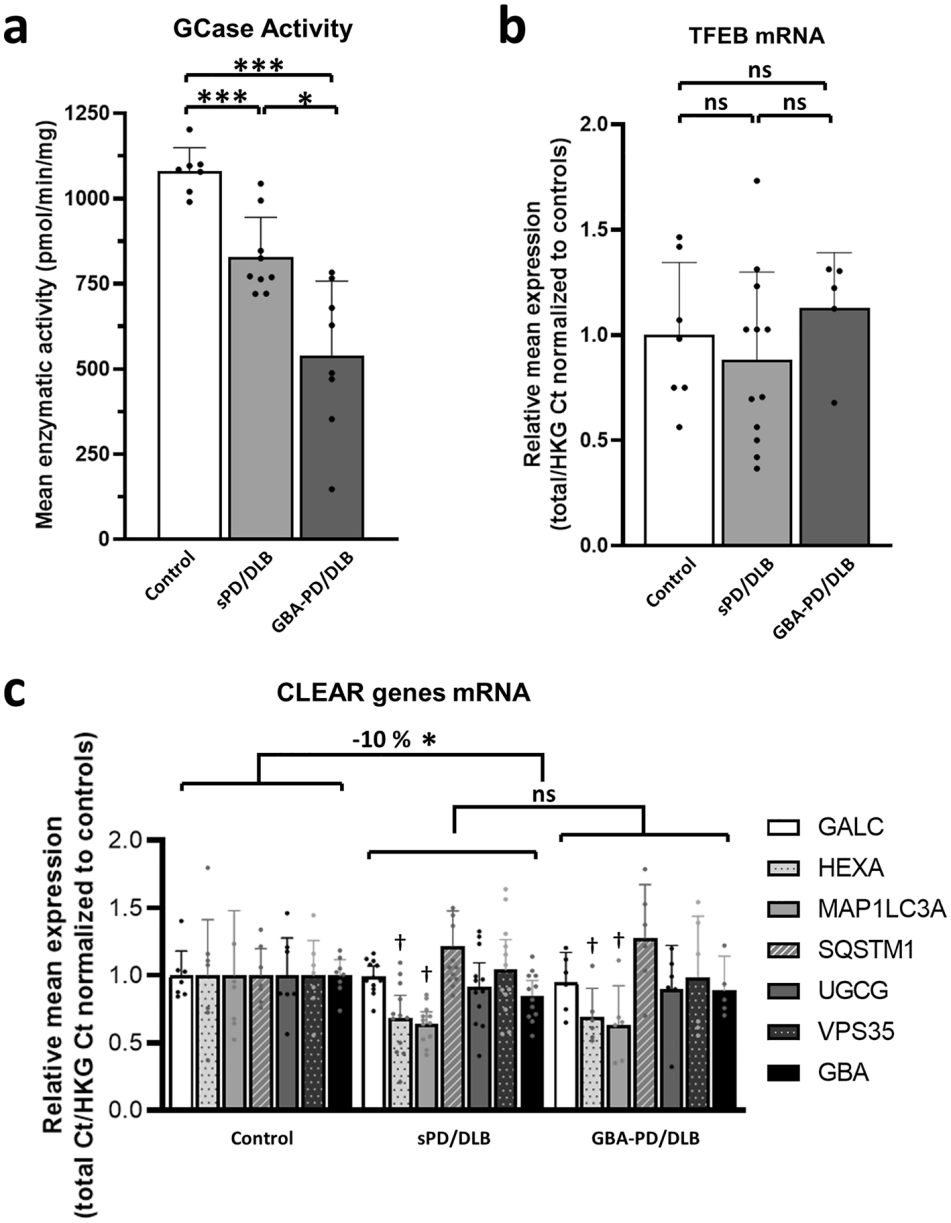
Impaired TFEB localization is associated with a reduction of total GCase activity and expression of selected CLEAR genes in the SN of sPD/DLB and *GBA*-PD/DLB patients. **a **Total GCase enzymatic activity quantification in bulk SN tissue from sPD/DLB, *GBA*-PD/DLB patients and controls as measured in [39] and expressed as pmol/min/mg of total protein. Median ± 95% CI; N ≥ 7/group, *n* = 3. **b**–**c** mRNA quantification by qPCR in sPD/DLB, *GBA*-PD/DLB patients and controls calculated as total Ct normalized on HKGs and expressed as fold-change compared to the control group. **b **Quantification of TFEB mRNA. Mean ± SD; N ≥ 5/group, *n* = 3. **c **CLEAR genes mRNA quantification. A 10% overall reduction in mRNA expression in the selected genes is observed when comparing the diseased group (sPD/DLB + *GBA*-PD/DLB) to controls. Mean ± SD. N ≥ 5/group, *n* = 3. HKG: housekeeping genes; GALC: galactosylceramidase; HEXA: hexosaminidase subunit alpha; GBA: ß-glucocerebrosidase; MAP1LC3A: microtubule associated protein 1 light chain 3; SQSTM1: sequestosome 1; UGCG: UDP-glucose ceramide glucosyltransferase; VPS35: retromer complex component. *p < 0.05; ***p < 0.001; †*p* < 0.05 vs Control

We further investigated how cytoplasmic retention and clustering of TFEB in the diseased groups associated with the enzymatic activity of the lysosomal hydrolase GCase. We previously reported a decreased GCase activity in the SN of sPD/DLB and—more pronounced—of *GBA*-PD/DLB patients compared to controls in the same cohort [39]. In the subset of PD/DLB cases selected for this study, GCase activity was significantly decreased in patients compared to controls (mean difference = − 253.4; *p* = 0.0003; 95% CI [− 376.0: − 130.8]), and it was further reduced in *GBA*-PD/DLB compared to sPD/DLB (mean difference = − 275.2; *p* = 0.012; 95% CI [− 486.4: − 64.0]) (Fig. 5a).

To study whether the observed deregulation of TFEB could be associated with alterations in its transcription levels, we quantified TFEB mRNA by qPCR in sporadic and *GBA*-related PD/DLB and controls. Results did not show a statistically significant difference in the expression of the TFEB gene in the disease groups compared to controls (Fig. 5b). Similarly, we quantified the expression of selected genes belonging to the CLEAR gene network which are under the transcriptional regulation of TFEB [56]. We observed a modest (10%) but statistically significant collective reduction in the expression of the selected CLEAR genes in the disease patients compared to control (*p* = 0.048) (Fig. 5c). This suggests a downregulation of the CLEAR gene network in the SN of patients with PD/DLB. Data obtained in MFG material showed a similar pattern (Suppl. Figure 7, online resource). No statistically significant difference was shown when testing for differences between individual diagnostic groups. Among the tested genes, MAP1LC3A showed reduction in mRNA expression in the sPD/DLB (*p* = 0.027; Wald Chi-Square: 4.899; *B* = 0.366; 95% CI [0.042: 0.691]) and *GBA*-PD/DLB (*p* = 0.012; Wald Chi-Square: 6.384; *B* = 0.357; 95% CI [0.080: 0.635]) group compared to controls. For HEXA (*p* = 0.017; Wald Chi-Square: 5.715; *B* = 0.313; 95% CI [0.056: 0.571]) and *GBA* (p = 0.044; Wald Chi-Square: 4.065; *B* = 0.140; 95% CI [0.004: 0.275]), mRNA expression was found to be decreased only when comparing diseased patients grouped against controls (Fig. 5c).

### TFEB cluster score correlates with GCase activity and Braak LB stage

**Fig. 6:**
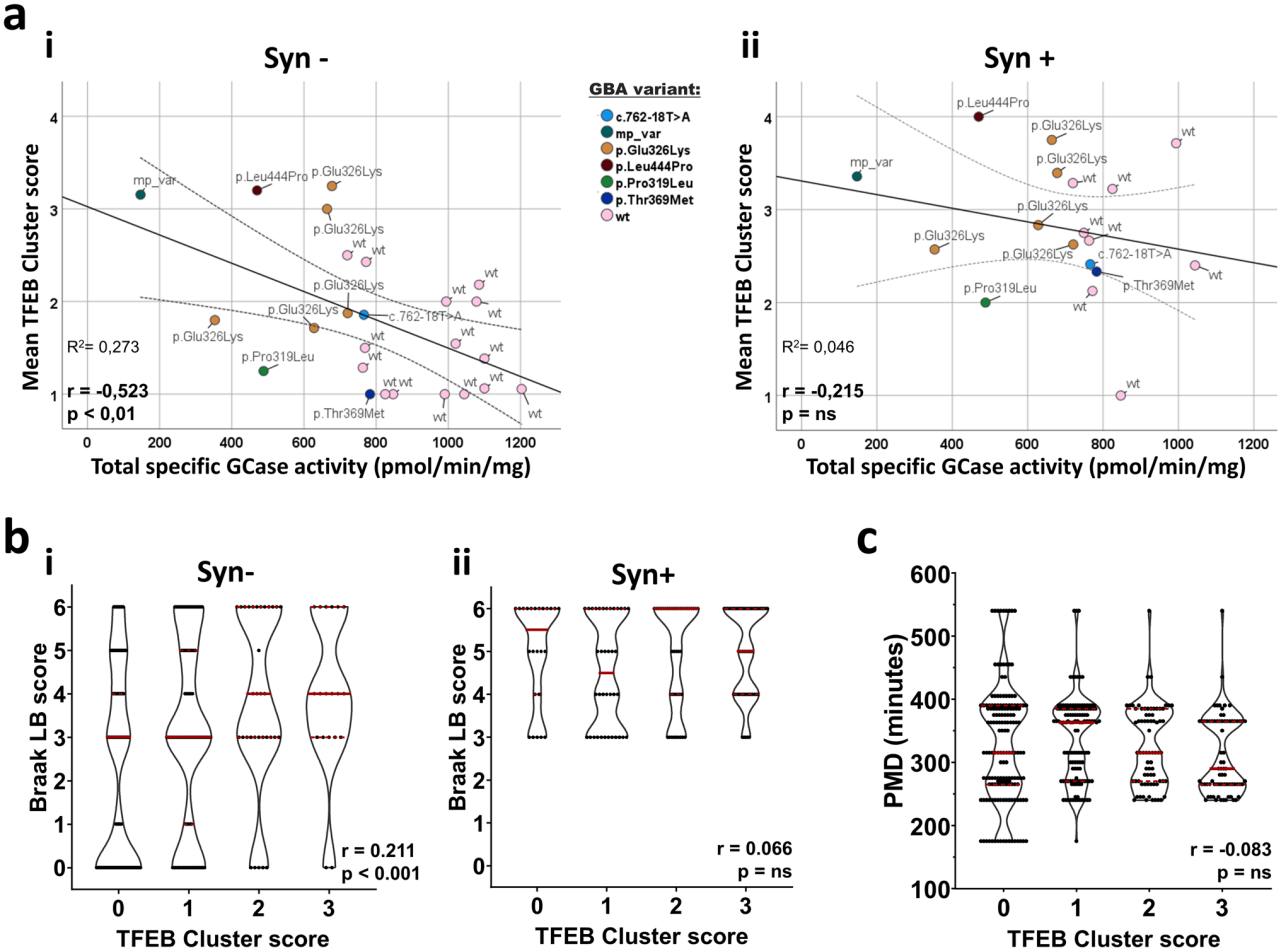
TFEB cluster score correlates with GCase enzymatic activity and with disease progression, as defined by Braak LB staging. **a** Pearson’s correlation analysis between mean TFEB cluster score and total GCase enzymatic activity from bulk SN tissue (previously measured in [39]). Wild-type (wt) and *GBA* mutation-carrier cases are color-coded as indicated in the graph (mp_var = multiple variants). Dotted lines indicate ± mean 95% confidence interval. A negative correlation between the readouts is observed (*r* = − 0.523, *p* < 0.01) in aSynuclein-negative (Syn-) cells (**a**-**i**). No significant correlation is observed in aSynuclein-positive (Syn +) cells (*r* = − 0.215, *p* = n.s.) (**a**-**ii**). **b** Frequency distribution of TFEB cluster score and Braak LB disease stages, as a measure of disease progression. Spearman’s correlation analysis reveals a statistically-significant positive correlation between the scores when analyzing Syn- cells (**b**-**i**) (*r* = 0.211, *p* < 0.001). The association is not significant in Syn + cells (**b**-**ii**) (*r* = 0.066, *p* = n.s.). **c** Frequency distribution of *post-mortem* delay (PMD) and TFEB cluster score. Spearman’s correlation analysis reveals no statistically-significant correlation between PMD and TFEB cluster scores (*r* = − 0.083, *p* = n.s.)

As TFEB is a master regulator of GCase expression [44, 49, 60], we hypothesized that the observed cytoplasmic retention and clustering of TFEB could be associated with changes in total GCase enzymatic activity. To assess this, we conducted a Spearman’s correlation analysis between total GCase activity in SN tissue lysate and mean TFEB cluster score per case (Fig. 6a). The analysis revealed a strong negative correlation between the two measurements (*r* = − 0.523, *p* < 0.01) in aSyn-negative cells, with cells from cases with lower total GCase activity showing higher TFEB cluster scores (Fig. 6a-i). *GBA*-PD/DLB cases had lower total GCase activity and showed higher amount of TFEB clustering compared to wild-type *GBA* cases (sPD/PDD and controls), which had lower cluster scores. Remarkably, the case (ID24) bearing three *GBA* variants (p.Asp140His, p.Glu326Lys, and p.Thr369Met) showed the lowest total GCase enzymatic activity and very high mean TFEB cluster score. No significant correlation between total GCase enzymatic activity and mean TFEB cluster score was observed in aSyn-positive cells (*r* = − 0.215, *p* = 0.428.) (Fig. 6a-ii). When testing for the difference in the regression lines of the correlations between TFEB cluster score and GCase activity between aSyn-positive or aSyn-negative cells, we identified a difference in the regression constant (y intercept; *B* = 0.862, *p* < 0.001), but not in regression slope (*p* = 0.408). Correlation analysis between mean TFEB nuclear score and total SN GCase activity in aSyn-negative cells identified a modest positive correlation which was not significant (Spearman’s correlation, *r* = 0.213 *p* = 0.305) (Suppl. Figure 8a, online resource).

To study the association between the measured TFEB cluster score and Braak LB stage, we analyzed their correlation in all cases studied (Fig. 6b). Higher Braak LB stage was associated with higher amount of TFEB clusters in aSyn-negative cells (*r* = 0.211, *p* < 0.001) (Fig. 6b-i). The same effect was not observed when analyzing only aSyn-positive cells (*r* = 0.066, *p* = n.s.) (Fig. 6b-ii. This suggests that TFEB clustering might associate with disease progression. We observed a modest positive trend in the correlation between TFEB cluster score and disease duration, which was not significant (*r* = 0.110, *p* = 0.061). Moreover, the observation of TFEB clusters in cells which have not yet developed detectable pSer129 aSyn cytopathology, could indicate the involvement of TFEB in the early stages of aSyn accumulation.

To exclude the possibility that the observed TFEB clustering pattern could be influenced by differences in *post-mortem* delay (PMD), we analyzed the association between the TFEB score and PMD (Fig. 6c), but found no significant correlation (Spearman’s correlation, *r* = − 0.083, *p* = n.s.). Similar analysis for nuclear TFEB has identified a modest but significant negative correlation between higher semi-quantitative scores and PMD (Spearman’s correlation, *r* = − 0.105, *p* = 0.014) (Suppl. Figure 8e, online resource). This indicates that part of the observed effect of TFEB nuclear translocation by semi-quantitative scoring can be explained by differences in PMD between subjects. Nonetheless, no significant difference in PMD between the groups was observed.

## Discussion

A growing number of pathological studies have focused on dissecting the determinants of ALP impairment in synucleinopathies. While several of these studies revealed mild effects on different ALP components in the context of PD/DLB, these results are sometimes contradictory [37, 72]. In the present study, we demonstrated that alterations in the subcellular localization of TFEB, master regulator of ALP, in nigral neuromelanin-containing neurons is strongly associated with PD and DLB in *post-mortem* human SN tissue. In line with earlier studies, we observed less nuclear localization of TFEB in nigral dopaminergic neurons of sPD/DLB and *GBA*-PD/DLB donors compared to control subjects. The increased cytoplasmic retention of TFEB was associated with its clustering at the Golgi apparatus. Interestingly, these effects were also observed in iLBD (Braak LB stage ≤ 4). This finding might indicate that altered TFEB localization initiates during the early stages of intracellular aSyn deposition. Nonetheless, this consideration needs to be taken carefully given the limitations of *post-mortem* studies in investigating temporal succession of events. Moreover, while we made the assumption that iLBD patients represent an early stage of Lewy body disease, (part of) these cases may represent alternative pathways. Due to age difference between iLBD and the other groups (Table 1), the effect observed in iLBD might be partially driven by age. Our small sample size did not allow insight into sex differences. Moreover, TFEB deregulation was accompanied by an overall reduction in CLEAR genes mRNA expression levels and GCase enzymatic activity in bulk tissue, indicating that the TFEB impairment might result in, or stem from, alterations of the ALP. In support of this, the observed effects were more pronounced in the *GBA*-PD/DLB group compared to sPD/DLB.

At the cellular level, TFEB clustering was notably increased in cells without detectable pSer129 aSyn cytopathology in *GBA* carriers and iLBD cases compared to controls. Moreover, the clustering was more severe in late pathological stages (as defined by Braak LB stage) and was increased in neurons with intracellular pSer129 aSyn deposition. While this finding may suggest that, at the cellular level, cytoplasmic TFEB clustering could happen prior to pathological aSyn accumulation, it is possible that early-stage aSyn aggregation—not detected by our pSer129 aSyn-based approach—might have already initiated in (a subset of) these cells. While the specific aSyn proteoforms that mark the earliest cellular stages of PD remain unidentified, recently-developed assays, including proximity ligation assay, offer promising methods for detecting early pathological aSyn conformers, such as oligomers [55, 59]. Although these methods still need validation in larger patient cohorts, they may represent an interesting approach for future studies.

In 2013, Decressac et al. showed that adeno-associated virus (AAV) vector-mediated overexpression of human wild-type aSyn impairs TFEB in the midbrain [15]. This was reflected by dynamic changes in CLEAR gene products (both mRNA and protein expression levels) over-time and by decreased TFEB protein levels in nuclear *versus* cytoplasmic tissue fractions, as demonstrated by Western blot [15]. In addition, decreased nuclear TFEB immunoreactivity was observed in sPD patients compared to controls. In our study, we aimed at reproducing and expanding on this finding in PD/DLB cases with and without *GBA*-mutations, as well as in iLBD cases. We employed multiple labeling experiments and high-resolution microscopy to allow for a deeper insight into the detailed subcellular localization of TFEB and its relation to the presence of subcellular pSer129 aSyn pathology. In line with the result of Decressac et al. [15], we observed significantly reduced nuclear TFEB immunoreactivity in SNpc dopaminergic neurons and an overall reduction of CLEAR genes expression levels in the midbrain of patients with sPD/DLB and, particularly, with *GBA*-PD/DLB. The differences observed between the sporadic and *GBA*-related group could be ascribed to the existence of a more acute TFEB phenotype caused by mutations in *GBA*. These mutations result in reduced GCase activity and have been previously associated with ALP impairment [28, 42, 58, 69]. In accordance, the subcellular immunoreactivity patterns of the two patients with severe *GBA* variants and low residual GCase activity [39]—one donor carrying the pathogenic L444P variant and one donor with three *GBA* variants (p.Asp140His, p.Glu326Lys, and p.Thr369Met)—were characterized by reduced nuclear labelling and extensive TFEB clusters. These clusters were shown to occupy a substantial volume of the Golgi apparatus (Figs. 2, 3), both in cells with and without aSyn pathology.

Apart from PD, reduced expression of TFEB in nuclear tissue fractions has been reported in different neurodegenerative diseases, including Alzheimer’s disease and Amyotrophic lateral sclerosis [12, 76]. These results demonstrate that nuclear TFEB localization is reduced in different neurodegenerative diseases in which protein aggregation takes place, thus suggesting that impaired nuclear translocation of TFEB might be related to aberrant protein homeostasis.

Besides neuronal cytoplasmic and nuclear TFEB punctae, we observed larger perinuclear immunopositive clusters colocalizing with Golgi markers. A link between TFEB and the Golgi apparatus was previously established in gene ontology analyses of the CLEAR network, showing an important role for TFEB in the transcriptional control of elements of the Golgi apparatus [44]. Moreover, recent evidence has suggested a role of the Golgi apparatus in ALP functioning in early stages of autophagy, amongst others in mannose 6-phosphate receptors-mediated sorting of lysosomal enzymes [10], as a source of membranes of double-layered membranes for ALP components [13, 21], and for the formation of autophagosomes [24]. Together, these findings suggest that the Golgi system might be a regulatory hub for the TFEB pathway.

Further investigation is warranted to determine the relevance of the unusual localization of TFEB at the Golgi observed in this study. Importantly, TFEB does not possess an ER signal sequence and does not enter the ER-Golgi protein maturation pathway during translation. In accordance, the observed TFEB immunoreactivity was not localized at the Golgi lumen, but at the membrane level (Fig. 3). Since TFEB is not a membrane-bound protein, its clustering might be due to protein–protein interaction at the cytosolic membrane side. Physiologically, TFEB interacts with lysosomes via the binding of its regulator mTORC1. This protein complex forms at the cytosolic membrane side of the lysosome thanks to the interaction of its components with the membrane-anchored lysosomal protein Rheb, among others. This leads to the activation of mTORC1, which retains and inactivates TFEB [31]. Recent literature has demonstrated strong localization of mTOR at the Golgi apparatus and has established it as a site for mTOR activation [22]. Moreover, Hao et al. [25] identified a significant non-canonical pool of Rheb protein which localizes at the Golgi, and can activate mTORC1 at lysosome-Golgi contact-points. Golgi-localized Rheb might function as an anchoring and activation site for the formation of the mTOR complex and the retention of TFEB at this site. Furthermore, it has been demonstrated that the induction of cellular stress by starvation, which has been shown to lead to the activation of a TFEB-mediated response [60], promotes a dynein-dependent retrograde transport of lysosomes to the Golgi complex [11, 77]. Similarly, perinuclear clustering of lysosomes localized at the Golgi have been observed in response to starvation [68]. The description of a pool of active Rheb and mTOR protein at the Golgi level, and the existence of a stress-induced association of lysosomes with the Golgi, might explain the retention of TFEB at the Golgi, as identified in this study. In line with this, we observed signs of cellular stress in neurons displaying perinuclear TFEB clusters, such as abnormalities in nuclear and cellular morphology. Nonetheless, we did not observe significant TFEB pattern differences in hESC-derived neurons between *GBA* KO and WT lines, which might be due to the need for longer differentiation time to manifest the *GBA*-related phenotype, or because of cell selection and compensatory mechanisms during culturing that favor mutation-insensitive cells. Future research could benefit from employing a GCase inhibitor in WT cells or a *GBA* conditional knockdown line to directly investigate *GBA* deficiency's impact on TFEB distribution. Albeit future experiments are thus necessary, our observations indicate that the Golgi apparatus might be an important regulatory center for TFEB function, particularly in the presence of proteolytic stressors such as pSer129 aSyn pathology.

Another important question that remains to be addressed is whether the TFEB present in the observed clusters is phosphorylated. Unfortunately, using currently available antibodies against phosphorylated TFEB, we were unable to obtain specific immunostaining in human postmortem brain samples by IHC. The development of sensitive and specific tools for visualizing phosphorylated TFEB in the human brain are needed to address this question.

Previous studies demonstrated a high structural homology between aSyn and 14–3-3 proteins, a phospho-serine binders and a negative regulators of TFEB [40, 43, 52, 79]. This family of proteins has been shown to regulate cellular response to stress and nutrient availability pathways [48]. This includes the inhibition of common negative regulators of mTORC1, such as TSC2 and PRAS40. aSyn has been shown to co-immunoprecipitate with 14–3-3 proteins and to interact with proteins which are common binders of 14–3-3 proteins [43]. In the present study, we observed negligible colocalization of aSyn with TFEB. Nonetheless, Decressac et al*.* previously reported the identification of colocalization between TFEB and aSyn within classical (ring-shaped) LBs [15]. In contrast, we consistently observed the lack of colocalization between TFEB and aSyn in diverse (non-LB) aSyn morphologies using antibodies against different aSyn epitopes (Fig. 4; Suppl. Figure 6, online resource). Nevertheless, we observed punctate TFEB patterns, specifically, localized at the periphery of classical ‘ring-shaped’ LB. This observation was unique to this aSyn morphology and might be attributed to the increased presence of lysosomes in the external layer of classical LBs, as previously described [62].

**Fig. 7:**
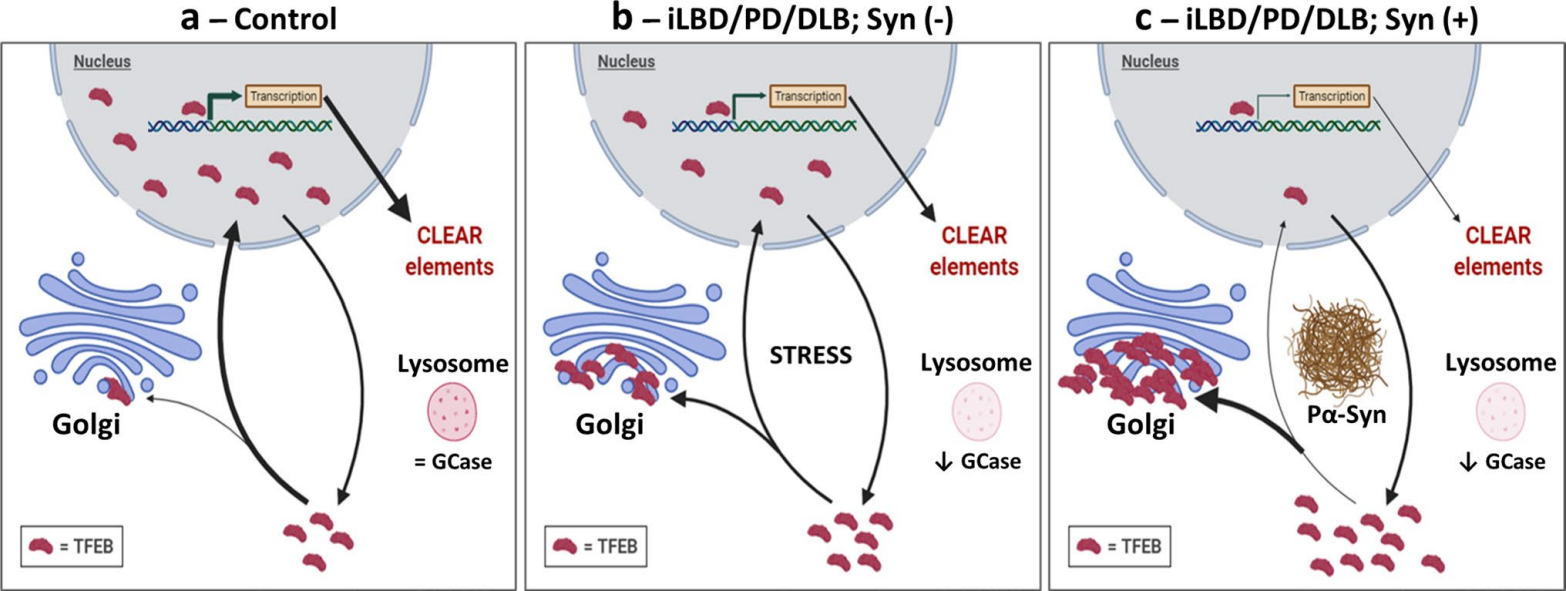
Hypothetical model of early TFEB redistribution and clustering in relation to pSer129 aSyn accumulation in PD/DLB neuronal soma. In dopaminergic neurons from the SN of control cases, physiological localization of TFEB is preserved, which results in the physiological transcription of CLEAR network genes necessary for the correct function of the autophagy and lysosomal pathway (panel **a**). In PD/DLB and iLBD neurons, nuclear TFEB is reduced and TFEB clusters begin to form at the Golgi network in neurons prior to the apparent accumulation of pathological pSer129 aSyn (Pα-Syn) in the soma, possibly in response to cellular stress triggered by a reduction in GCase activity and resulting lysosomal impairment. The observed TFEB redistribution is associated with an overall reduction of the transcription of CLEAR elements (panel **b**). Neurons with apparent aSyn cytopathology present more severe TFEB clustering at the Golgi and a reduced nuclear pool (panel **c**) compared to PD/DLB neurons without intracellular aSyn (panel **b**). The observation that alterations of TFEB distribution occurs in pSer129 aSyn (-) cells, both in *GBA*-related and sporadic PD/DLB cases, as well as in iLBD donors, indicates a possible role for TFEB in the early disease mechanisms, possibly prior pSer129 aSyn accumulation in the soma

The observation that TFEB clusters in cells without detectable intracellular pSer129 aSyn deposition, and the increase in the fraction of cluster-positive cells observed both in iLBD and PD/DLB compared to controls in pSer129 aSyn-negative cells, may indicate that TFEB clustering is associated with early events during cellular disease progression. The positive correlation between Braak LB stage and TFEB clustering score observed in aSyn-negative cells suggests that the clustering might aggravate during disease progression (Fig. 6). Furthermore, the observation that TFEB clustering negatively correlates with total GCase activity, especially in the more severe phenotypes such as the *GBA*-PD/PDD group, suggests that cytoplasmic retention and clustering of TFEB could be triggered by lysosomal dysfunction. Based on these observations, we speculate that TFEB clustering might be an early disease-related process occurring prior to detectable pSer129 aSyn deposition in the soma (Fig. 7). Further mechanistic investigation is needed to validate this hypothesis. In particular, elucidating the functional significance and the mechanisms involved in the translocation of TFEB at the Golgi might provide further insights into potential early disease mechanisms and lead to the identification of a potential therapeutic window.

In conclusion, our results on TFEB subcellular localization in postmortem human brain tissue have demonstrated a link between TFEB deregulation and the development of *GBA*-related and sporadic PD/DLB. This suggests the potential involvement of TFEB in the early stages of the molecular development of PD/DLB, possibly prior to pathological pSer129 aSyn accumulation in the soma. Our observations support an important role for TFEB in the pathogenesis PD/DLB, thereby confirming the potential of targeting TFEB as a possible approach for urgently-required disease-modifying therapies for synucleinopathies.

### Supplementary Information

Below is the link to the electronic supplementary material.

Supplementary file1 (AVI 80.0 MB)

Supplementary file2 (PDF 2766 KB)

## Data Availability

All data supporting the findings of this study are available from the corresponding author upon request.
