# Supplementary material for: Altered TFEB subcellular localization in nigral neurons of subjects with incidental, sporadic and GBA-related Lewy body diseases
Source: Acta Neuropathol. 2024 Apr 6;147(1):67. doi: 10.1007/s00401-024-02707-z (PMC10998821; doi:10.1007/s00401-024-02707-z)
Supplement: Supplementary file 2 — Supplementary file2 (PDF 2766 KB) [file 401_2024_2707_MOESM2_ESM.pdf]

Electronic Supplementary Material

Altered TFEB subcellular localization in nigral neurons of subjects with incidental, sporadic and *GBA*-related Lewy body diseases

Tim E Moors[1,2] & Martino L Morella[1,2], Cesc Bertran-Cobo[1,2], Hanneke Geut[1,2], Vinod Udayar[3], Evelien Timmermans-Huisman[1,2], Angela MT Ingrassia[1,2], John JP Brevé[1,2], John GJM Bol[1,2], Vincenzo Bonifati[4], Ravi Jagasia[3], Wilma DJ van de Berg[1,2,†]

Affiliations:

[1] Section Clinical Neuroanatomy and Biobanking, Department of Anatomy and Neurosciences, Amsterdam UMC, Vrije University, Amsterdam, The Netherlands

[2] Amsterdam Neuroscience, program Neurodegeneration, Amsterdam, The Netherlands

[3] Roche Pharma Research and Early Development; Neuroscience and Rare Diseases Discovery and Translational Area; Roche Innovation Center, Basel, Switzerland.

[4] Erasmus MC, Department of Clinical Genetics, University Medical Center Rotterdam, Department of Clinical Genetics, Rotterdam, The Netherlands.

† Corresponding author:
Wilma D.J. van de Berg, PhD
Section Clinical Neuroanatomy and Biobanking, Department of Anatomy and Neurosciences, Amsterdam UMC, Vrije University, Amsterdam, The Netherlands
e-mail: wdj.vandeberg@amsterdamumc.nl

Supplementary Material and Methods

Western Blotting

Total cell lysate from human monocyte cell line HP1 was prepared in SDS sample buffer (final: 50 mM Tris-HCl, 10% glycerol, 2% SDS, 1% 2-mercaptoethanol, 12.5 mM EDTA, 0.02 % bromophenol blue) containing 50 mM DTT and then heated for 10 minutes at 95°C. Samples containing 40 µg of total protein were loaded per each well of a 10% acrylamide (29:1) TRIS/Glycine SDS gels (1.5 mm/10-well) and run at 150V for 1-1.5 hr. Proteins where than transferred on nitrocellulose membrane in Transfer Buffer (25mM Tris, 200mM glycine) containing 10% methanol. After blotting, the membranes were briefly washed in TBS-T (50 mM Tris, 150 mM NaCl, pH 7.6 + 0.1% Tween 20) and blocked for 30 min at RT in Odyssey blocking buffer:TBS (1:1)(Li-Cor). Blots were then cut in vertical strips and each was incubated with different TFEB antibodies diluted in Blocking buffer (Odyssey blocking buffer:TBS=1:1) or in blocking buffer alone (negative controls) and incubated overnight at 4°C. The following TFEB antibodies were used (for details, see Suppl. Tab. 2, online resource): TFEB A303-673A (Bethyl), TFEB ab220695 (Abcam), TFEB HPA049532 (Atlas Antibodies), TFEB MBS120432 (MyBioSource), TFEB ab270614 (Abcam), Phospho-TFEB (Ser122) #86843 (Cell Signaling Technology), TFEB ab2636 (Abcam). Dilutions were not optimized for the assay and are based on pre-existing literature. After primary, blots where washed in TBS-T and incubated with secondary antibodies (Goat anti-Rabbit, IDRye800CW, 1:10000, Li-cor 926-32211 or Donkey anti-Mouse IRDye800CW 1:10000, Li-cor 926-32212) diluted in Blocking buffer for 1 hr at RT. After washing with TBS-T and rinsing once in TBS, the strips were imaged together using Odyssey SA scanner and Image Studio Software (Li-Cor).

Sequential multiplex IHC

Post-mortem FFPE brain section were processed as described in the *Immunohistochemical staining procedure* section of Materials and methods until and including the antigen retrieval step. After, the slides were quenched in 3% hydrogen peroxide in TBS for 30 minutes at RT and then washed three times for 5 minutes in TBS and incubated in Blocking buffer (3% normal goat serum in TBS) for 30 minutes

at RT. The sections were than incubated with TFEB A303-673A (Rabbit, 1:100, Bethyl) primary antibody in Blocking buffer over-night at 4°C. In the morning, the sections were washed three times in TBS and incubated with Envision HRP anti-Rabbit (DAKO #K4003) for 1 hr at RT. After washing twice with TBS and once with 1M Tris-HCl, the slides were incubated with Alexa Fluor 594 Tyramide Reagent (Invitrogen #B40957) for 15 mins at RT and immediately washed 2 times in 1M Tris-HCl and once in TBS. In order to remove the antibodies from the tissue sections (stripping) and proceed with a sequential multiplex staining, the tissue sections were processed for antigen retrieval as previously described. The slides were than incubated with an anti-GOLGA2 primary antibody (Rabbit, 1:400 , HPA021230, Atlas Antibodies) in Blocking buffer for 4 hr at RT and then incubated with a directly-labelled secondary antibody (Goat anti-Rabbit Alexa Fluor™ 647, 1:400, Thermo Fisher #A-21245) in Blocking buffer containing 1 µg/ml of DAPI for 2 hr at RT. After washing, sections were mounted in Mowiol mounting solution using glass cover slips (Art. No.: 630-2746; Glaswarenfabrik Karl Hecht, Sondheim, Germany).

Differentiation of human embryonic stem cells to neurons

-Human embryonic stem cells

Human embryonic stem cells (hESCs) were obtained with ethical approval under Swiss research registry (BAG-hES-IMP-0031) from the UK Stem Cell Bank (UKSCB) (Steering comm. appl. SCSC07-14). Cell line source: Human Embryonic Stem Cell Line SA001, Cellartis AB; NIH Human Embryonic Stem Cell Registry no. 0085; origin info: male, ethnicity and age N/A. *GBA* KO was generated from the same hESC line as previously reported (*GBA*1_hESC_*GBA*#1−/−_10.18.11; identifier: CEBe033-A-1) [3].

-Neural precursor cells

Neural precursor cells (NPCs) were generated from human embryonic stem cells (hESC) according to a previously published protocol [1]. In brief, hESCs were plated as single cell suspension in AggreWell-800 (STEMCELL Technologies, #34815) to generate embryoid bodies (EBs) and maintained in NPC1 media. NPC1 media was composed of a DMEM/F-12-Neurobasal mix medium [1:1 mix of DMEM/F-12 medium

supplemented with 1% GlutaMax (Thermo Fisher #31331093) and neurobasal medium (Thermo Fisher #21103049) supplemented with 2% B27 supplement, 2% N2 supplement, and 50 µM 2-mercaptoethanol (Thermo Fisher #12587010, #17502048, #31350010 respectively)] supplemented with 5 ng/ml FGF-2, 250 ng/ml noggin (R&D Systems or Peprotech) and 20 µM SB 431542 (Tocris #1614) for 5 days. EBs were than plated in plates coated with polyornithine-laminin (PL) and maintained in the same medium for 3 day to form neural rosettes. At day 4, neural rosettes were manually dissociated and plated in PL-coated plates in NPC1. After reaching confluence, cells were dissociated and plated at 100 000 cells/cm^2 on PL-coated plates in NPC2 media (DMEM/F-12-Neurobasal mix medium supplemented with 10 ng/ml FGF-2 (Peprotech), 10 ng/ml EGF (R&D Technologies), and 20 ng/ml BDNF (Peprotech). Cells were then passaged every 2-3 days for about 15 times with a gradual decrease in cell density to 25 000 cells/cm^2 over the first 10 passages and maintained in NPC2 media with daily media changes.

-Differentiation to neuronal cells

Differentiation of NPCs to neurons was performed according to a previously-published protocol [2]. NPCs were plated at 10 000 cells/ cm^2 in PL-coated flasks and cultured for one week in DMEM/F-12-Neurobasal mix medium supplemented with 100 ng/ml FGF-8 (Peprotech), 200 ng/ml sonic hedgehog (Peprotech), and 100 µM ascorbic acid 2-phosphate (Sigma). Cells were then re-plated at 50 000 cells/cm^2 in neurobasal medium supplemented with 20 ng/ml BDNF, 10 ng/ml glial cell-derived neurotrophic factor (GDNF; Peprotech), 500 µM dibutyryl cyclic AMP (Sigma), and 100 µM ascorbic acid 2-phosphate and differentiated for 42 days before assays.

Supplementary Figures and Tables

Supplementary Table 1: Donors selected for this study. More detailed demographics were provided in Moors *et al*., 2017 [4].

**ID (ID in [4])**	**Pathological diagnosis**	**Age**	**Sex**	***GBA* variant**	**Severe *GBA* variant (Y/N)**	***GBA* variant classification**	**Braak LB stage**	**Braak NFT score**	**CERAD amyloid score**	**IHC exp.**	**GCase activity exp.**	**qPCR Exp.**	**Thal phase**
**1 (1)**	control	77	F	-	-	none	0	1	B	x	x	-	2
**2 (2)**	control	84	F	-	-	none	0	1	O	-	-	x	0
**4 (4)**	control	79	F	-	-	none	0	1	O	-	-	x	0
**5 (5)**	control	83	F	-	-	none	0	1	B	-	x	-	2
**6 (6)**	control	76	F	-	-	none	0	2	O	x	x	x	0
**7 (7)**	control	76	M	n.a.	n.a.	n.a.	0	0	O	-	-	x	1
**8 (8)**	control	70	F	-	-	none	0	2	A	x	x	x	1
**9 (9)**	control	83	F	-	-	none	0	2	B	x	-	-	n.a.
**10 (10)**	control	70	F	-	-	none	0	2	A	x	x	-	2
**12 (12)**	control	78	F	-	-	none	0	2	A	x	x	-	2
**14 (14)**	control	79	M	-	-	none	0	2	A	-	-	x	3
**15 (15)**	control	83	M	-	-	none	1	1	A	x	x	x	1
**17 (17)**	PD	81	F	-	-	none	5	0	O	x	x	-	0
**19 (19)**	PD	74	M	-	-	none	5	1	O	x	x	-	0
**22 (22)**	PD	81	M	-	-	none	5	1	O	x	x	x	0
**23 (23)**	PD	80	M	-	-	none	6	2	B	-	-	x	2
**25 (25)**	PD	72	M	-	-	none	6	1	A	x	x	-	3
**27 (27)**	PD	78	M	-	-	none	4	0	A	x	x	x	n.a.
**28 (28)**	PD	73	M	-	-	none	4	3	O	-	-	x	0
**29 (29)**	PD	81	F	-	-	none	6	3	A	-	-	x	n.a.
**30 (30)**	PD	76	M	-	-	none	6	2	O	-	-	x	0
**34 (34)**	DLB	70	M	-	-	none	6	1	A	-	-	x	3
**35 (35)**	DLB	83	M	-	-	none	5	2	B	-	-	x	n.a.
**36 (36)**	DLB	78	M	-	-	none	6	2	C	-	-	x	2
**39 (39)**	DLB	75	M	-	-	none	6	2	B	x	x	-	3
**41 (41)**	DLB	78	F	-	-	none	6	2	B	x	x	x	n.a.
**44 (44)**	DLB	83	M	-	-	none	6	0	O	x	x	x	0
**45 (45)**	DLB	74	M	-	-	none	4	1	B	x	x	x	2
**16 (16)**	PD	80	F	c.762-18T>A	n.a.	n.a.	6	1	C	x	x	-	1
**18 (18)**	PD	84	M	p.Pro319Leu	n.a.	n.a.	5	1	A	x	x	-	2
**21 (21)**	PD	76	M	p.Glu326Lys	N	Risk	6	1	O	x	x	x	0

**24 (24)**	PD	83	F	p.Asp140His, p.Glu326Lys, and p.Thr369Met	Y	Severe	4	1	O	x	x	x	0
**26 (26)**	PD	65	M	p.Leu444Pro	Y	Severe	6	1	B	x	x	x	3
**37 (37)**	DLB	72	M	p.Glu326Lys	N	Risk	6	1	O	x	x	-	0
**38 (38)**	DLB	80	F	p.Glu326Lys	N	Risk	5	1	O	x	x	x	0
**42 (42)**	DLB	81	F	p.Thr369Met	N	Risk	4	1	A	x	x	x	2
**43 (43)**	DLB	69	M	p.Glu326Lys	N	Risk	6	3	B	x	x	-	4
**50**	iLBD	93	F	-	-	none	3	2	O	x	-	-	0
**51**	iLBD	86	F	-	-	none	3	3	A	x	-	-	1
**52**	iLBD	98	M	-	-	none	3	2	O	x	-	-	0

Supplementary Table 2: Details of the antibodies used in the present study. All antibodies were tested for IHC with the following antigen retrieval (AR) methods: 30 min Heat Induced Epitope Retrieval (HIER) in 10 mM Citrate Buffer pH 7,0 (HIER-CB); 30 min HIER in 10mM Tris-EDTA buffer pH 9.0; 10 min in 100% Formic Acid (FA); HIER-CB + FA; 10 min 1 units/mL Proteinase K in Tris-EDTA buffer pH 8.0 at 37°C. Successful AR methods used in this study are reported for each antibody.

Reference/ Art no.	RRID	Source	Epitope	Host (clonality)	Dilution (µg/ml)	Antigen Retrieval IHC
11A5	n.a.	Prothena (Gift)	P–Ser129 α–syn	Mouse (monoclonal)	IHC: 1:20000 (0,3) WB: -	CB-HIER or CB-HIER + FA
MABN389 (5G4)	AB_2716647	Merck Millipore	α–syn (aa 46-53)	Mouse (monoclonal)	IHC: 1:800 (n.a.) WB: -	CB-HIER + FA
#610786 (Clone 42, SYN1)	AB_398107	BD Biosciences	α–syn (aa 91-99)	Mouse (monoclonal)	IHC: 1:100 (2,5) WB: -	CB-HIER + FA
ab27766 (LB 509)	AB_727020	Abcam	α–syn (aa 115-122)	Mouse (monoclonal)	IHC: 1:300 (3,33) WB: -	CB-HIER + FA
sc-7012 (N-19)	AB_634977	Santa Cruz	α–syn (aa 1-60)	Goat (polyclonal)	IHC: 1:100 (1) WB: -	CB-HIER
A303-673A	AB_11204751	Bethyl	TFEB	Rabbit (polyclonal)	IHC: 1:100 (10) WB: 1:1000 (1)	CB-HIER
ab220695	n.a.	Abcam	TFEB	Rabbit (polyclonal)	IHC: 1:25 (n.a.) WB: 1:100 (n.a.)	CB-HIER
HPA049532	AB_2680807	Atlas Antibodies	TFEB	Rabbit (polyclonal)	IHC: no signal WB: 1:170 (15,26)	-

MBS120432	AB_2271743	MyBioSource	TFEB	Mouse (monoclonal)	IHC: no signal WB: 1:100 (1)	-
ab270614	n.a.	Abcam	TFEB	Rabbit (monoclonal)	IHC: no signal WB: 1:1000 (0,98)	-
ab2636	AB_303224	Abcam	TFEB	Goat (polyclonal)	IHC: no signal WB: 1:500 (0,5)	-
#86843	AB_2890188	Cell Signaling Technology	Phospho-TFEB (Ser122)	Rabbit (polyclonal)	IHC: no signal WB: 1:1000 (0,005)	-
PA5-114662	AB_2899298	Thermo Fisher Scientific	Phospho-TFEB (Ser211)	Rabbit (polyclonal)	IHC: no signal WB: 1:1000	-
#37681	AB_2799117	Cell Signaling Technology	Phospho-TFEB (Ser211)	Rabbit (monoclonal)	IHC: no signal WB: -	-
ABE1971-I	AB_2928101	Merck Millipore	Phospho-TFEB (Ser142)	Rabbit (polyclonal)	IHC: no signal WB: -	-
60004-1-Ig	AB_2107436	Proteintech	GAPDH	Mouse (monoclonal)	IHC: - WB: 1:5000 (0,2)	-
NBP1-49643	AB_10011762	Novus Biologicals	TGN46	Rabbit (polyclonal)	IHC: 1:150 (6,67) WB:-	CB-HIER
#610524	AB_397884	BD Biosciences	Calnexin	Mouse (Monoclonal)	IHC: 1:25 (10) WB: -	CB-HIER
HPA021230	AB_1849839	Atlas Antibodies	GOLGA2	Rabbit (polyclonal)	IHC: 1:100 (7) WB: -	CB-HIER
H4B4	AB_528129	Developmental Studies	LAMP-2	Mouse (monoclonal)	IHC: 1:200 (n.a.) WB: -	CB-HIER

		Hybridoma Bank				
MAB422	AB_95101	EMD Millipore	Cathepsin D	Mouse (monoclonal)	IHC: 1:100 (10)<br>WB: -	CB-HIER

Supplementary Table 3: Number of nigral neuromelanin-containing dopaminergic neurons with and without pathology scanned in the different diagnostic groups for the semi-quantitative analysis of TFEB

		Pathology		
		**α-SYN (–)**	**α-SYN (+)**	**Total**
**Diagnosis**	**Control**	98	0	98
	**iLBD**	74	31	105
	**sPD/DLB**	54	55	109
	***GBA*-PD/DLB**	57	72	129
	**Total**	283	158	**441**

Supplementary Table 4: List of primers used in the qPCR experiments

Target	Primer sequence<br>5' 3'	UPL probe number	Holding stage (95°C)	Denaturation (95°C)	Annealing (T(°C)/sec)	Extension (65°C)
HEXA	F: ACACTTCCGCTGTGAATTGCTG<br>R: GCTCCACTACCATTCACCTACA	# 15	15 min	15 sec	59 °C/30 sec	40 sec
MAP1LC3A	F: GACCATGTCAACATGAGCGAG<br>R: CGTAGACCATATAGAGGAAGCC	# 15	15 min	15 sec	59 °C/30 sec	45 sec
SOSTM1	F: GAAGCTGCCTTGTACCCACA<br>R: CCGATGTCATAGTTCTTGGTCTG	# 16	15 min	15 sec	60 °C/30 sec	55 sec
UGCG	F: CAAAGCGATAGCTGACCGAG<br>R: CTGGCAACAAAGCATTCTGAAATTG	# 4	15 min	15 sec	59 °C/30 sec	45 sec
VPS35	F: CTCTTCTGGTCTGGCAGAAAC<br>R: TCTTCTCGAATCTTTTGGATAAGCTG	# 52	15 min	15 sec	60°C/30 sec	55 sec
GALC	F: GTGTGTTCATTGCAGGAAGAG<br>R: GTAACTTCAACACGTCCTAAAGC	# 6	15 min	15 sec	59 °C/30 sec	45 sec
GBA	F: CCTACTCATGCTGGATGACCA<br>R: CCAATGTACAGCAATGCCATGAA	# 43	15 min	15 sec	58 °C/35 sec	30 sec
TFEB	F: GAGATGACCAACAAGCAGCTC<br>R: AGCTCAGCCATGTTCATGCC	# 17	15 min	15 sec	56 °C/30 sec	30 sec
OAZ1	F: CACCATGCCGCTCCTAAG<br>R: ACAGCAGTGGAGGGAGAC	# 74	15 min	15 sec	57°C/30 sec	30 sec
POL2RF	F: CATGTCAGACAACGAGGACAA<br>R: TCCAAGTCATCTAGCCCTTCA	# 25	15 min	15 sec	60°C/30 sec	30 sec
POLR2A	F: CGCATCATGAACAGCGATGAG<br>R: GCAGGGTCATATCTGTCAGCAT	# 69	15 min	15 sec	59°C/30 sec	35 sec
PES1	F: AGATGCAGAGGCTGGTTCA<br>R: CTCACTCTCCTCCTCCT	# 21	15 min	15 sec	59°C/30 sec	30 sec

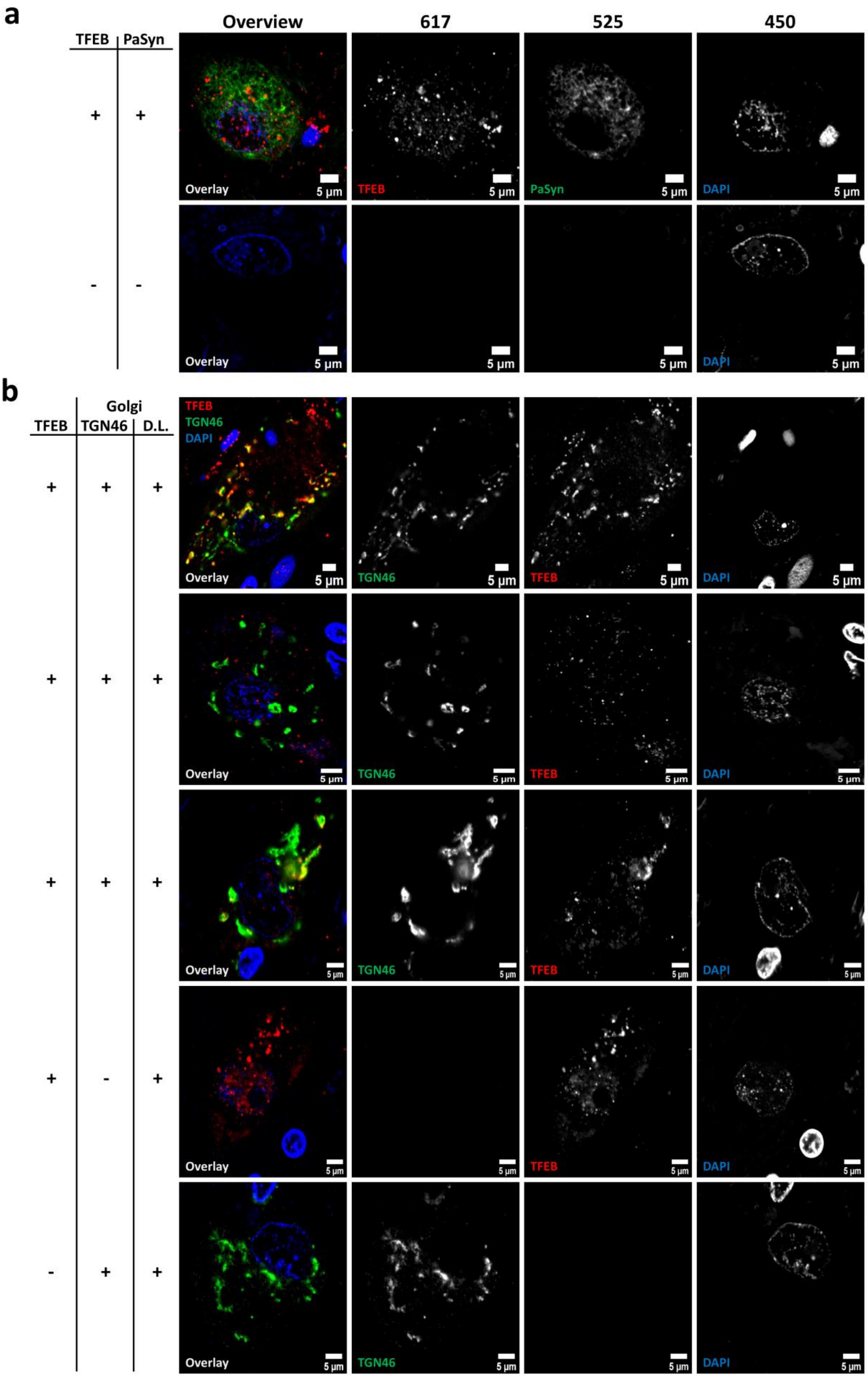
a
Overview
617
525
450
TFEB
PaSyn
+
+
-
-
Overlay
TFEB
PaSyn
DAPI
5 µm
b
Golgi
TFEB
TGN46
D.L.
TFEB
TGN46
DAPI
+
+
+
-
Overlay
TGN46
TFEB
DAPI
5 µm

Supplementary Figure 1: Representative negative control images for the primary antibodies used in the main IHC experiments.

Representative row confocal images of negative controls experiments for the main IHC experiments of this. Acquisitions are presented per each emission channel (617, 525, 450). **a:** Cells stained with (upper row) and without (lower row) anti-TFEB (Bethyl A303-673A, red) and anti-pSer129 aSyn (PaSyn, green) primary antibody. **b:** Cells stained with either anti-TFEB (Bethyl A303-673A, red) antibody and directly labelled (D.L.) anti-TGN46 (green) antibody (upper 3 rows), with the D.L. in absence of TGN46 primary antibody (4th row), or without TFEB primary antibody (5th row). Scale bar = 5 µm.

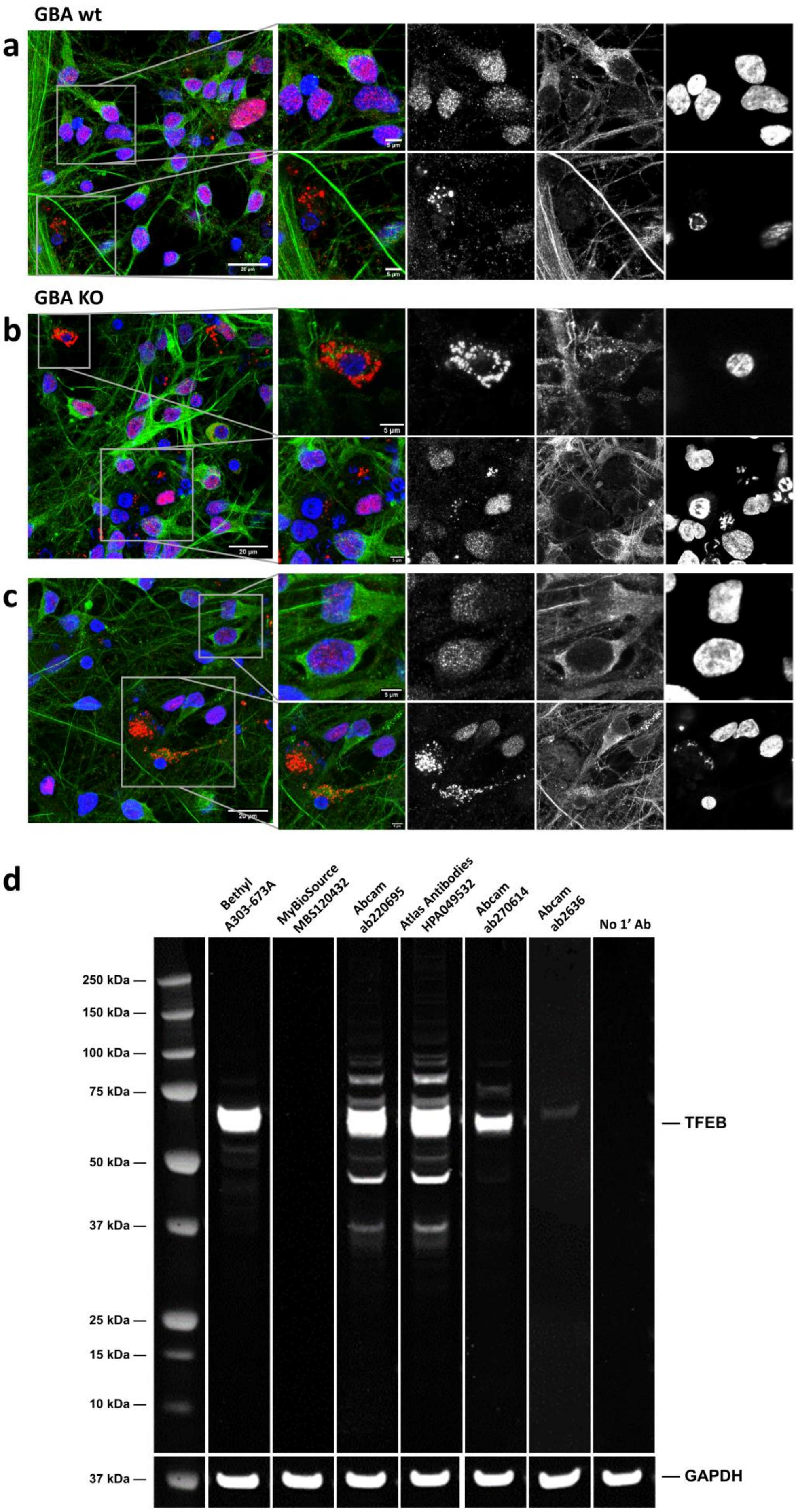

GBA wt
a
GBA KO
b
c
d
Bethyl A303-673A
MyBioSource MBS120432
Abcam ab220695
Atlas Antibodies HPA049532
Abcam ab270614
Abcam ab2636
No 1' Ab
250 kDa
150 kDa
100 kDa
75 kDa
50 kDa
37 kDa
25 kDa
15 kDa
10 kDa
37 kDa
TFEB
GAPDH

Supplementary Figure 2: Specificity of TFEB antibodies.

a-c: TFEB clusters are observed in human embryonic stem cell-derived neurons in vitro with TFEB A303-673A antibody. Representative raw confocal overview images (left panels) and zoom-ins (right panels) of hESC-derived isogenic dopaminergic neurons stained for TFEB (red) and MAP2 (green) showing TFEB perinuclear clusters similar of what observed human *post-mortem* brain. Cells displaying TFEB clusters were often displaying signs of cellular stress, such as altered cellular morphology, reduced nuclear shape and DNA condensation. **a:** *GBA* wild-type (wt) neurons. **b:** *GBA* knock-out (KO) neurons. No difference in the percentage of cluster-positive neurons could be observed among the two lines. Scale bar = 20/5 µm as indicated in each panel. TFEB antibody: Bethyl A303-673A. **d:** Comparison of the specificity of TFEB antibodies in HP1 cells lysate by western blot as indicated. See details in Table S2. Primary antibody were omitted in the negative control (No 1'Ab) lane.

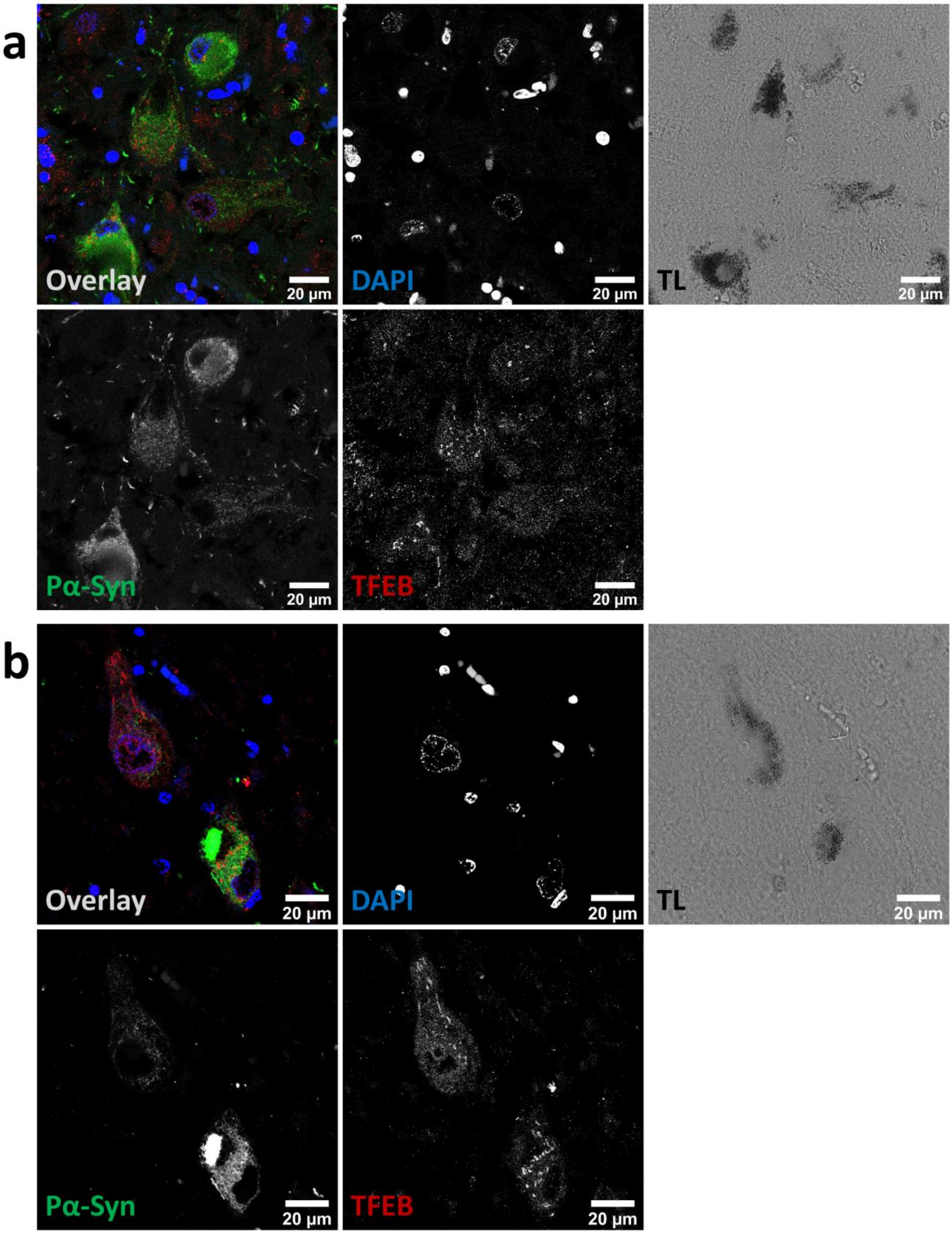

Supplementary Figure 3: Representative lower magnification fluorescent IHC images of TFEB clusters with the Bethyl A303-673A antibody.

Representative transmitted light (TL, right panels) and raw CLSM (other panels) images of SNpc from donor ID19 (sPD, **a**) and ID34 (sDLB, **b**). Staining for TFEB (red) and Ser129-phosphorylated

aSyn (Pα-Syn, green) reveals the presence of intracellular aSyn cytopathology and perinuclear TFEB clusters. Scale bar = 20 µm. TFEB antibody: Bethyl A303-673A.

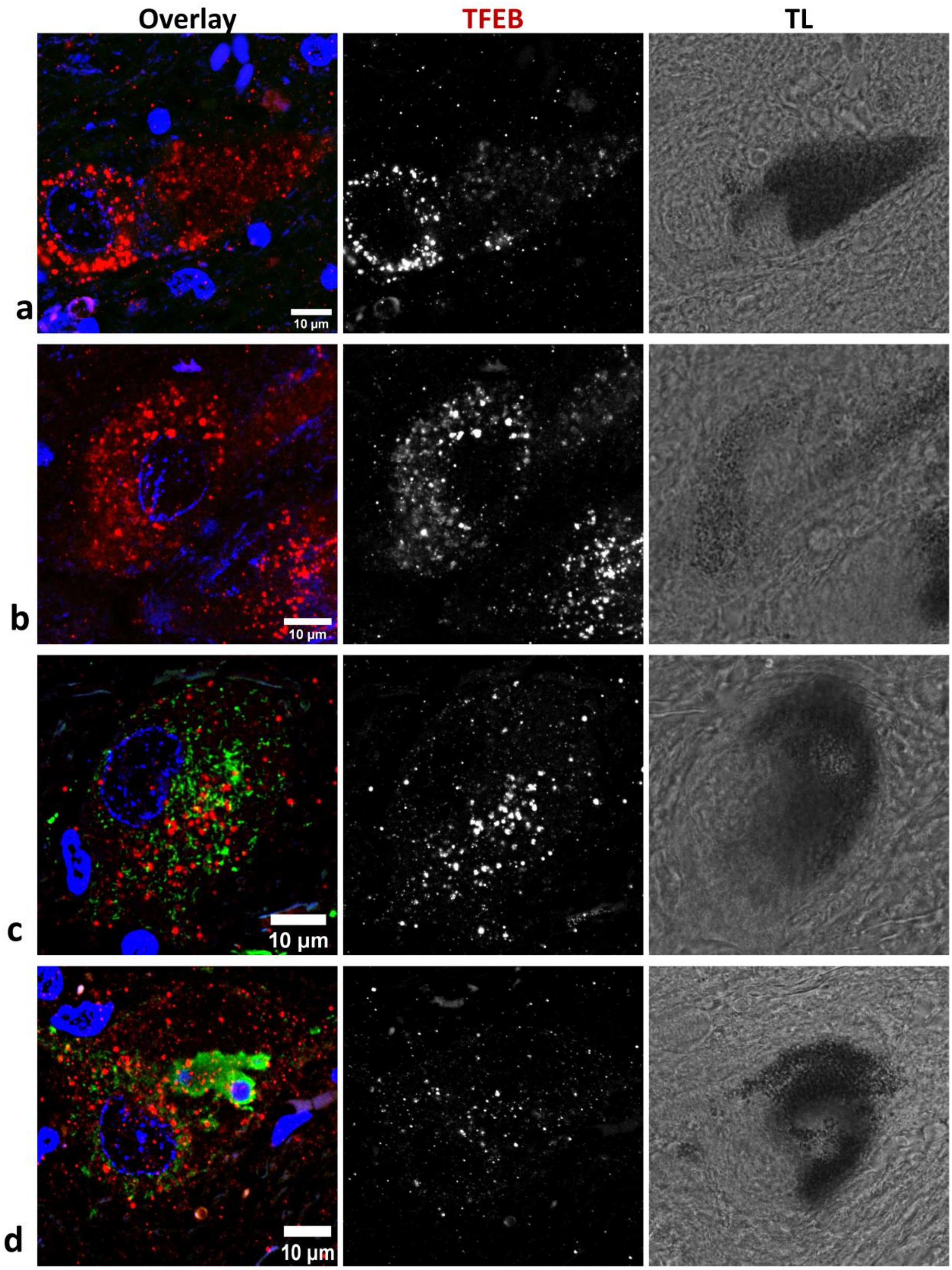

Supplementary Figure 4: Representative fluorescent IHC images of TFEB immunopositive clusters with Abcam ab220695 antibody.

Representative transmitted light (TL, right panels) and raw CLSM (other panels) images of SNpc from donor ID17 (sPD, **a**), ID39 (sDLB, **b-c**), ID25 (sPD, **d**) stained for TFEB (red) and Ser129-

phosphorylated aSyn (green) showing the presence of perinuclear TFEB positive clusters immunoreactive to Abcam ab220695 TFEB antibody. Scale bar = 10 µm. TFEB antibody: Abcam ab220695.

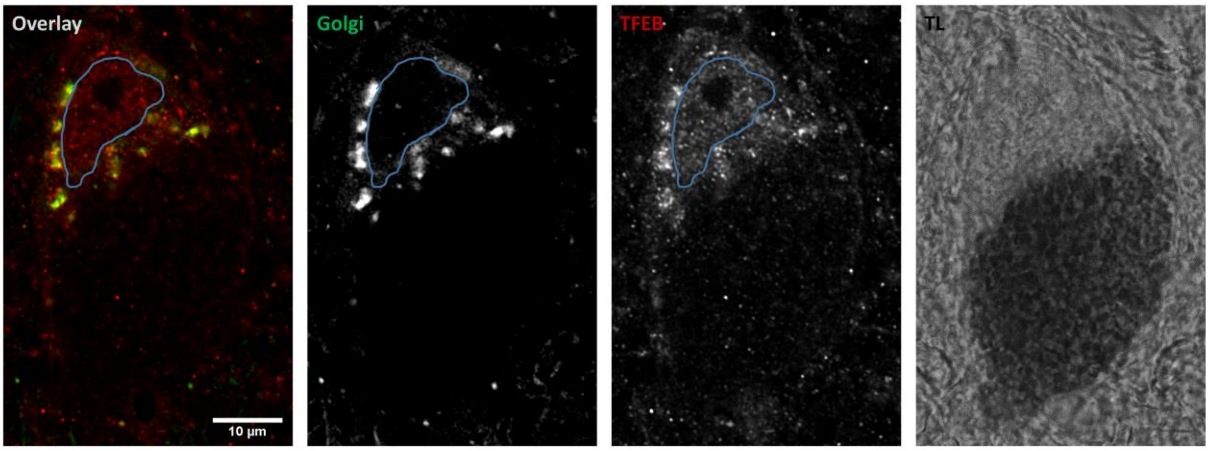

Supplementary Figure 5: Representative sequential fluorescent IHC image of TFEB immunopositive clusters localized at the Golgi.

Representative transmitted light (TL, right panel) and raw CLSM (other panels) images of SNpc from donor stained for TFEB (red) and the cis-Golgi marker GOLGA2 (green) stained with sequential fluorescent IHC as described in the methods sections showing the localization of perinuclear TFEB clusters at the Golgi. Donor ID26 (*GBA*-DLB).Scale bar = 10 µm. TFEB antibody: Bethyl A303-673A.

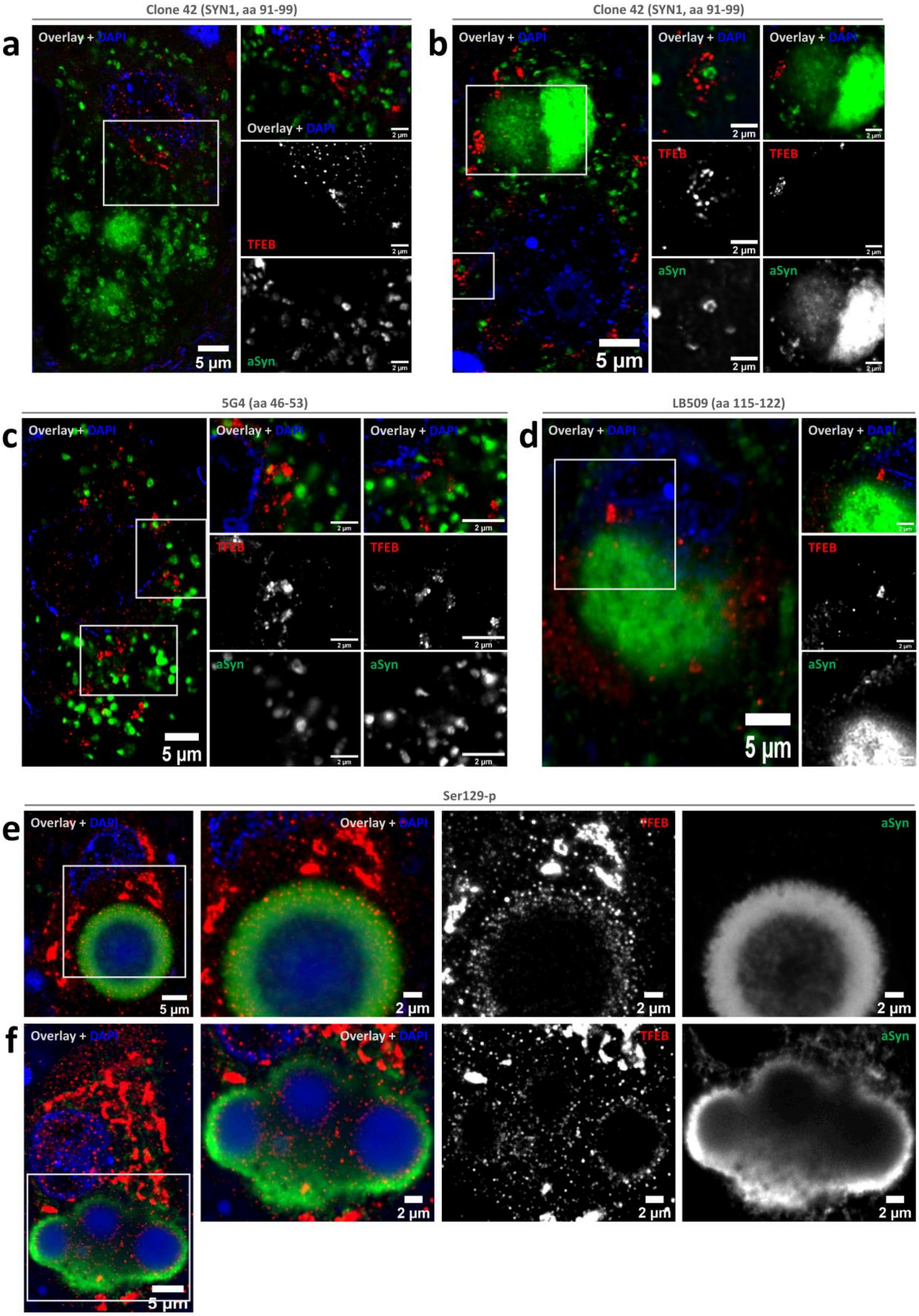

Supplementary Figure 6: TFEB clusters do not colocalize with aSyn detected with antibodies targeting different aSyn epitopes.

Representative deconvolved CLSM (left panels) and STED (zoom-ins) images of dopaminergic SNpc neuron from donor ID26 (*GBA*-DLB; **a,d,e**) and ID38 (*GBA*-DLB**; b,c,f**). Colocalization analysis of TFEB (red) and several anti-aSyn antibodies (green). **a-b:** Staining with Clone 42 (SYN1) aSyn antibody targeting aa91-99. **c:** Staining with 5G4 aSyn antibody targeting aa46-53. **d:** Staining with LB509 aSyn antibody targeting aa155-122. **e-f:** Staining with anti-pSer129 aSyn antibody (Ser129-p) targeting phosphorylated aSyn. No colocalization between TFEB clusters and aSyn was observed. Some colocalization was observed between TFEB punctae and aSyn within the outer layer of fully-formed onion-shaped Lewy bodies as identified with anti-pSer129 aSyn antibody (**e-f**). Scale bars = 5/2 uM µm as indicated in each panel. TFEB antibody: Bethyl A303-673A.

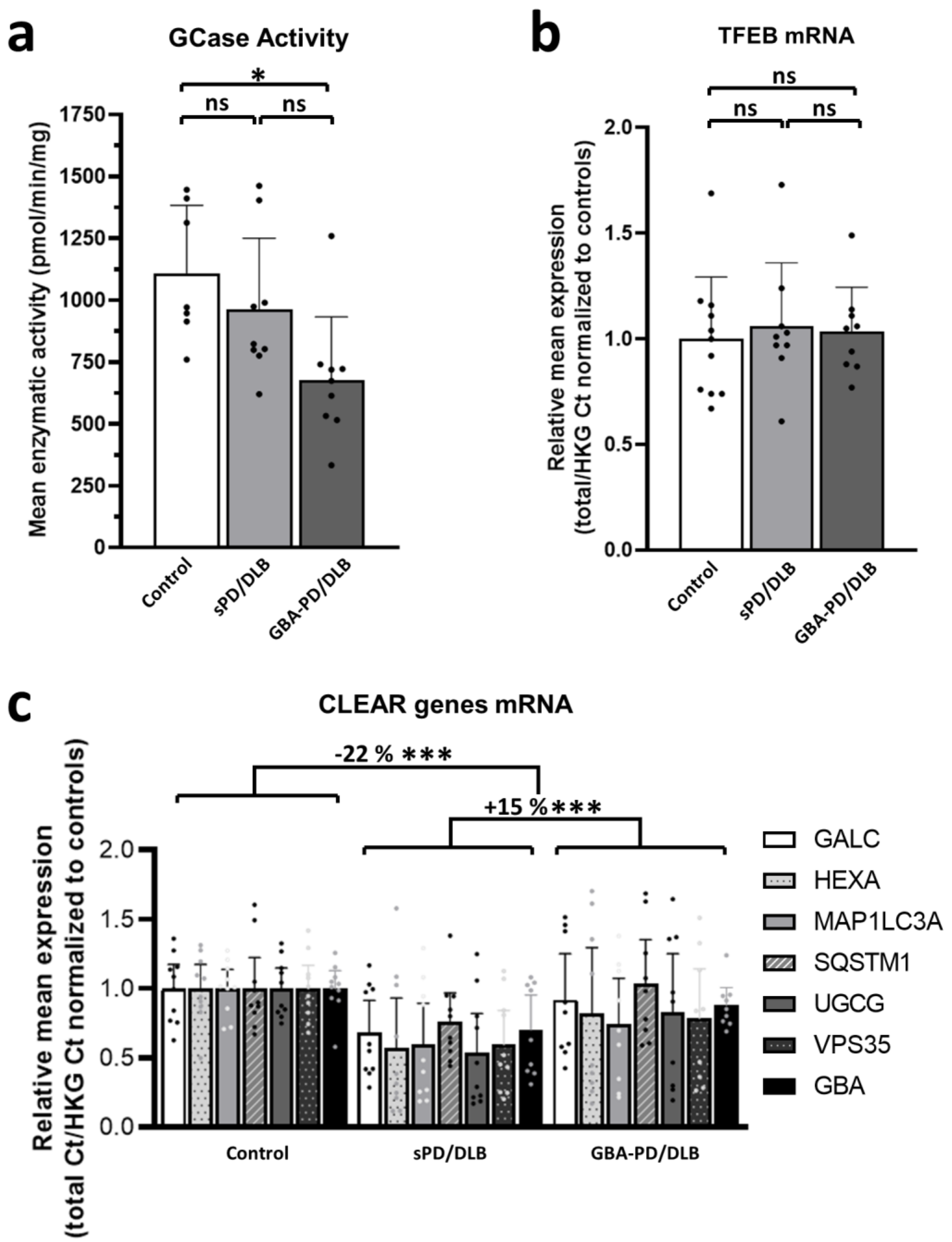

Supplementary Figure 7: Decreased GCase activity and selected CLEAR genes expression in the medial frontal gyrus of sPD-DLB and *GBA*-PD/DLB patients. **a:** Mean total GCase enzymatic activity quantification in bulk medial frontal gyrus tissue from sPD/DLB, *GBA*-PD/DLB patients and controls expressed as pmol/min/mg of total protein. Mean ± SD; N≥7/group, n=3. **b-c:** mRNA quantification by qPCR in sPD/DLB, *GBA*-PD/DLB patients and controls calculated as total Ct normalized on HKGs and expressed as fold-change compared to the control group. **b:** Quantification of TFEB mRNA. Mean ± SD; N≥5/group, n=3. **c:** CLEAR genes mRNA quantification. Mean ± SD. N≥5/group, n=3. HKG: housekeeping genes; GALC: galactosylceramidase; HEXA: hexosaminidase subunit alpha; GBA: ß-glucocerebrosidase; MAP1LC3A: microtubule associated protein 1 light chain 3; SQSTM1: sequestosome 1; UGCG: UDP-glucose ceramide glucosyltransferase; VSP35: retromer complex component. *p < 0.05; ***p < 0.001; † p < 0.05 vs Control.

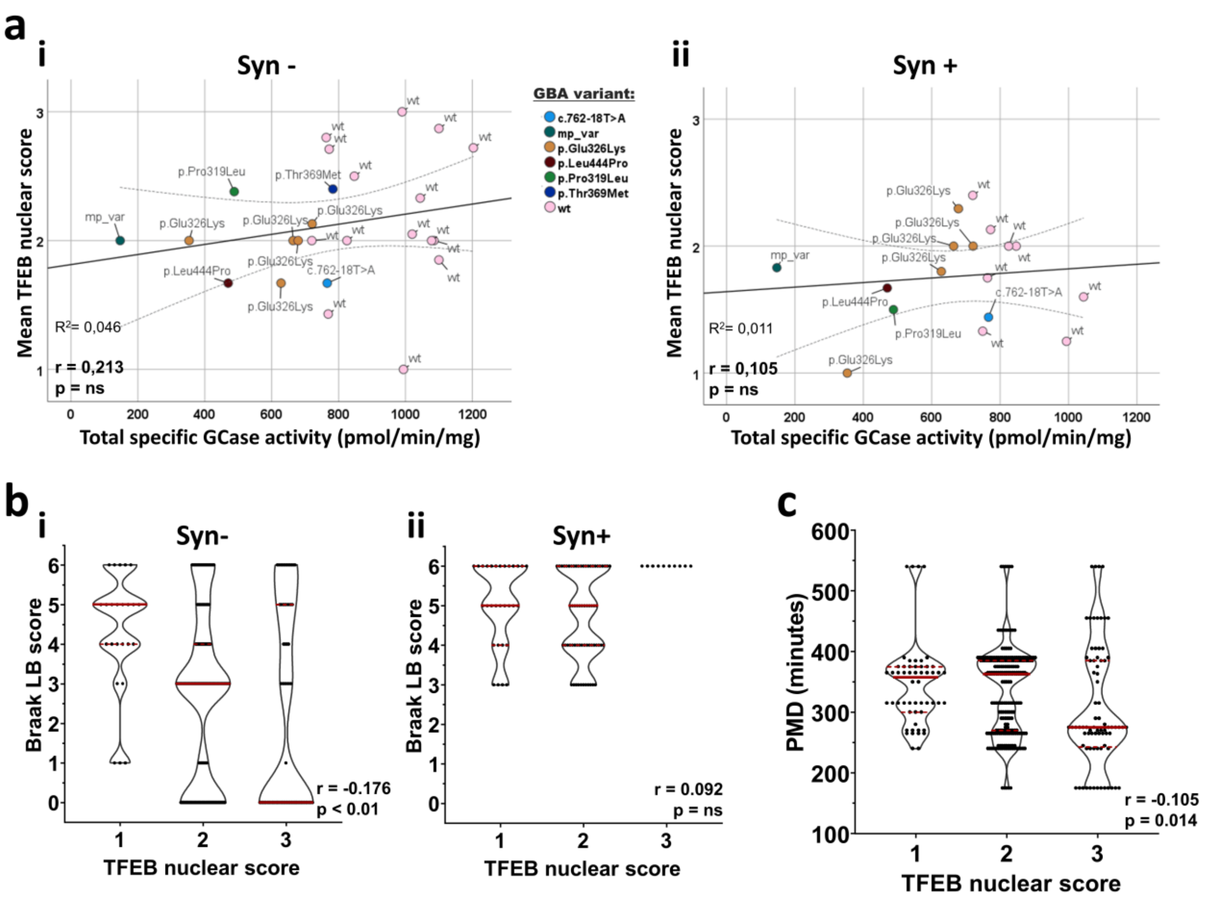

Supplementary Figure 8: TFEB nuclear score correlation with GCase enzymatic activity in the SN and with disease progression, as defined by Braak LB staging.

a: Pearson's correlation analysis between mean TFEB cluster score and total GCase enzymatic activity from bulk medial frontal gyrus tissue (previously measured in [4]). Wild-type (wt) and *GBA* mutation-carrier cases are color-coded as indicated in the graph (mp_var = multiple variants). Dotted lines indicate +/- mean 95% confidence interval. No statistically-significant correlation between the readouts was observed both in aSynuclein-negative (Syn-) cells (r= 0.213, p= ns) (**a-i**) and in aSynuclein-positive (Syn+) cells (r= 0.105, p= ns) (**a-ii**). **b:** Frequency distribution of TFEB cluster score and Braak LB disease stages, as a measure of disease progression. Spearman's correlation analysis revealed a statistically-significant positive correlation between the scores when analyzing Syn- cells (**b-i**) (r = -0.176, p < 0.01). The association was not significant in Syn+ cells (**b-ii**) (r = 0.092, p = ns). **c:** Frequency distribution of *post-mortem* delay (PMD) and TFEB cluster score. Spearman's correlation analysis reveals no statistically-significant correlation between PMD and TFEB cluster scores (r = -0.105, p = 0.014).

Supplementary Video 1: 3D surface render reconstruction of TFEB cluster around the nucleus in a dopaminergic SNpc neuron.

Representative 3D surface render reconstruction from CLSM image series of a dopaminergic SNpc neuron from donor ID38 (*GBA*-DLB) stained for TFEB (red), pSer129 aSyn (green) and DAPI (blue). The video shows the clustering of TFEB around the nucleus. The scale bar is indicated in the video. TFEB antibody: Bethyl A303-673A.

Supplementary References

1 Costa V, Aigner S, Vukcevic M, Sauter E, Behr K, Ebeling M, Dunkley T, Friedlein A, Zoffmann S, Meyer CAet al (2016) mTORC1 Inhibition Corrects Neurodevelopmental and Synaptic Alterations in a Human Stem Cell Model of Tuberous Sclerosis. Cell Rep 15: 86-95 Doi 10.1016/j.celrep.2016.02.090

2 Gehrlein A, Udayar V, Anastasi N, Morella ML, Ruf I, Brugger D, von der Mark S, Thoma R, Rufer A, Heer Det al (2023) Targeting neuronal lysosomal dysfunction caused by beta-glucocerebrosidase deficiency with an enzyme-based brain shuttle construct. Nat Commun 14: 2057 Doi 10.1038/s41467-023-37632-4

3 Gundner AL, Meyer CA, Aigner S, Christensen K, Patsch C, Jagasia R, Baumann K, Burcin M (2017) Generation of a homozygous GBA deletion human embryonic stem cell line. Stem Cell Res 23: 122-126 Doi 10.1016/j.scr.2017.07.009

4 Moors TE, Paciotti S, Ingrassia A, Quadri M, Breedveld G, Tasegian A, Chiasserini D, Eusebi P, Duran-Pacheco G, Kremer Tet al (2018) Characterization of Brain Lysosomal Activities in GBA-Related and Sporadic Parkinson's Disease and Dementia with Lewy Bodies. Mol Neurobiol: Doi 10.1007/s12035-018-1090-0
